# Supplementary material for: Expanding detection windows for discriminating single nucleotide variants using rationally designed DNA equalizer probes
Source: Nat Commun. 2020 Oct 29;11:5473. doi: 10.1038/s41467-020-19269-9 (PMC7596233; doi:10.1038/s41467-020-19269-9)
Supplement: Supplementary file 1 — Supplementary Information [file 41467_2020_19269_MOESM1_ESM.pdf]

Supplementary Materials

Expanding detection windows for discriminating single nucleotide variants using rationally designed DNA equalizer probes

Guan A. Wang,[1,2] Xiaoyu Xie,[2] Hayam Mansour,[2,3] Fangfang Chen,[1,2] Gabriela Matamoros,[4,5] Ana L. Sanchez[4,5] Chunhai Fan,[6] and Feng Li,*[,1,2]

[1]Key Laboratory of Green Chemistry & Technology of Ministry of Education, College of Chemistry, Sichuan University, Chengdu, Sichuan, China, 610064

[2]Department of Chemistry, Centre for Biotechnology, Brock University, St. Catharines, Ontario, Canada, L2S 3A1

[3]Department of Cell Biology, National Research Centre, Cairo, Egypt, 12622

[4]Department of Health Sciences, Brock University, St. Catharines, Ontario, Canada, L2S 3A1

[5]Microbiology Research Institute, National Autonomous University of Honduras (UNAH), Tegucigalpa, Honduras

[6]School of Chemistry and Chemical Engineering, Shanghai Jiao Tong University, Shanghai, 201240, China

*Corresponding author: Feng Li, windtalker_1205@scu.edu.cn

Table of Contents

1. Propagation of detection windows in toehold-exchange probs..3
2. Theoretical framework and mathematical simulation..4
3. Experimental validation and optimization of DEG..19
4. Effect of target and DEP length..27
5. Detection of varying single nucleotide mutations using DEG.......................................30
6. Evaluation of DEG for detection of rare mutations..43
7. Narrowing concentration ranges for quantifying dsDNA targets using DEG.....................44
8. DEG-PCR...45
9. Analyzing clinical parasitic worm specimens using DEG-PCR.....................................49
10. DNA sequences and modifications...58

1. Propagation of detection windows in toehold-exchange probes

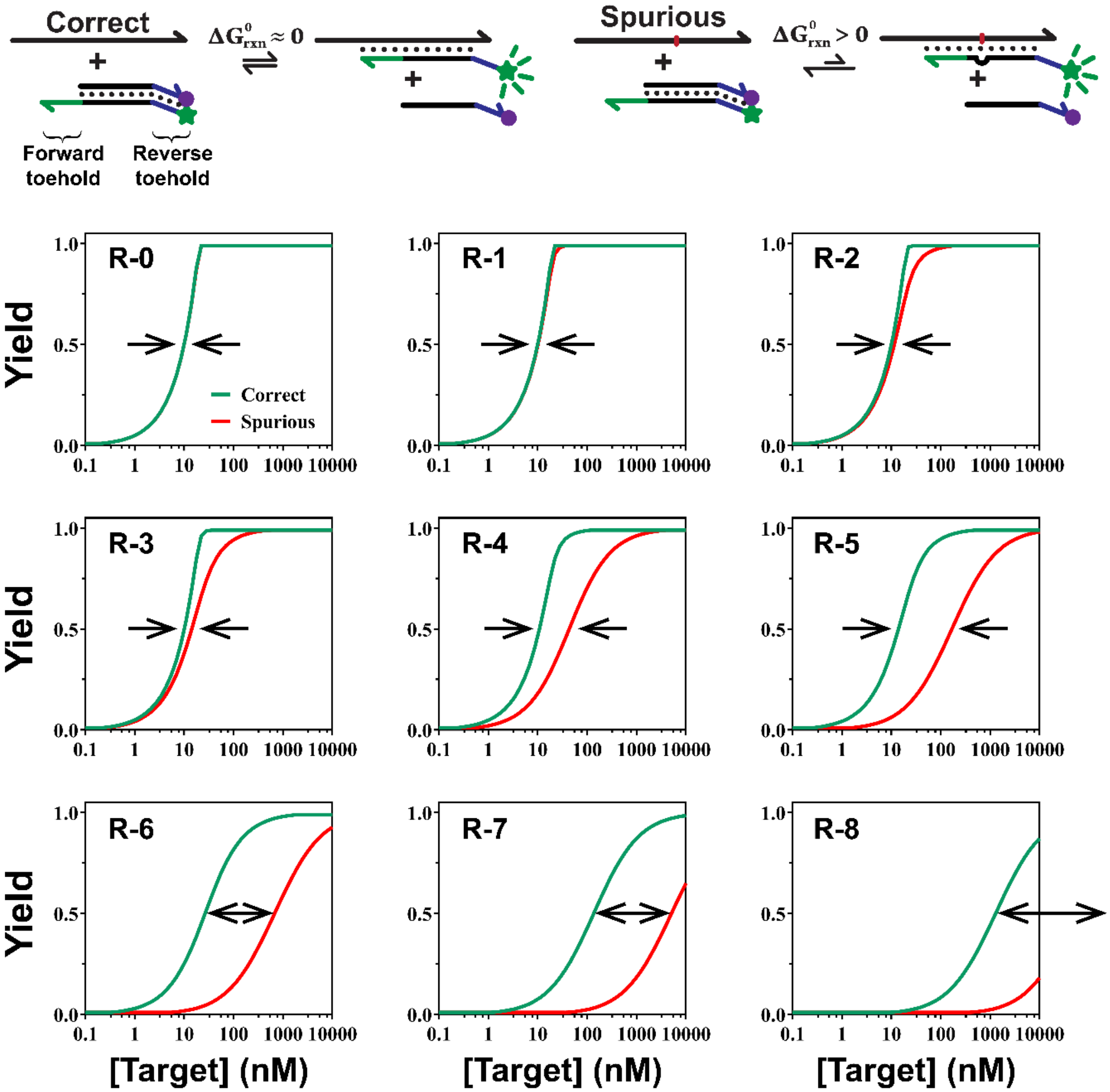

Supplementary Figure 1 | Propagation of detection windows in toehold-exchange probes. Simulation demonstrates that the detection windows (indicated by the arrows) for discriminating a pair of correct and spurious targets (ΔΔG = 2.28 kcal/mol) can be enlarged for by increasing energy barriers via the elongation of reverse toeholds of toehold-exchange probes (r = 0 nt to r = 8 nt). The length of forward toehold is fixed at 7 nt. The simulation also reveals that the enlarged detection window is also a cost of shifting the entire window to the higher concentration end.

2. Theoretical framework and mathematical simulation

The detection window reflects the performance of nucleic acid hybridization probes across wide concentration ranges. Concentration is a vital variable in evaluating the sensitivity and specificity of DNA hybridization probes, yet the concentration dependency of yield, discrimination factor (DF), and robustness factor (RF) remains unexplored in a systematic manner. We first set out to profile the concentration-dependency of hybridization yield and sequence specificity across a wide concentration range in silico. Toehold-exchange probes are chosen as a testbed in our system. To highlight the numerical relationship between variables, we applied a dimensionless transformation to all concentrations prior to derivations.

2.1 Concentration dependence and robustness

A toehold exchange reaction can be simplified to a bimolecular reversable reaction (eq. 1)[1].

$$T + CP \rightleftharpoons TC + P, with\ \Delta G^0_{rxn};\ \text{(eq. 1)}$$

where *T* is the target, *C* is the partial complementary strand to *T* and *P* is the protecting strand for *C*. Thermodynamics of a toehold-exchange probe is tunable by altering the lengths of forward and reverse toehold or by controlling the stoichiometry between *CP* and *P*. The free energy of each reactant and product can be calculated using NUPACK software.

Zhang and colleagues have previously defined the yield of the reaction as $\eta^* = \frac{[TC]}{\min([T]_0,[CP]_0)}$, where $[T]_0$ and $[CP]_0$ are the initial concentrations of *T* and *CP*, respectively.[2] We reason that this definition is useful for guiding the sequence design of toehold-exchange probes but not suitable for predicting the analytical behaviors of probes at specified experimental conditions, because the initial target concentration is often an unknown variable in the system. Practically, the concentration of *CP* is fixed, and *T* is a

variable, so we defined the reaction yield as $\eta = \frac{[TC]}{[CP]_0}$. We also chose $[CP]_0$ as the characteristic concentration for dimensionless transformations.

For a typical reversible reaction, the equilibrium constant can be derived from the reaction free energy ΔG^0 (eq. 2) and the concentration of all nucleic acid species follows the rule of mass conservation.

$$K_{eq} = e^{-\Delta G^0/RT} = \frac{[TC]_{eq}\cdot[P]_{eq}}{[T]_{eq}\cdot[CP]_{eq}} = \frac{[TC]_{eq}\cdot([P]_0+[TC]_{eq})}{([T]_0-[TC]_{eq})\cdot([CP]_0-[TC]_{eq})}; \text{(eq. 2)}$$

We further dimensionless the formula converting concentrations into numeric values, where the target concentration in its dimensionless form is denoted as τ and that of $[P]_0$ denoted as γ, respectively (eq. 3).

$$K_{eq} = e^{-\Delta G^0/RT} = \frac{[TC]_{eq}\cdot([P]_0+[TC]_{eq})/[CP]_0^2}{([T]_0-[TC]_{eq})\cdot([CP]_0-[TC]_{eq})/[CP]_0^2}$$

$$= \frac{\eta\cdot(\gamma+\eta)}{(\tau-\eta)\cdot(1-\eta)}; \text{(eq. 3)}$$

$$where\ \gamma \coloneqq {}^{[P]_0}\!/_{[CP]_0}\ and\ \tau \coloneqq {}^{[T]_0}\!/_{[CP]_0}\ respectively.$$

The concentration dependence of η is solved by equation S4:

$$\eta = \frac{K_{eq}+\gamma+K_{eq}\tau-\sqrt[2]{K_{eq}^2\tau^2-2K_{eq}^2\tau+K_{eq}^2+2K_{eq}\gamma\tau+2K_{eq}\gamma+4K_{eq}\tau+\gamma^2}}{2(K_{eq}-1)}$$

$$= \frac{(K_{eq}+\gamma+K_{eq}\tau)-\sqrt[2]{(K_{eq}+\gamma+K_{eq}\tau)^2-4K_{eq}\tau(K_{eq}-1)}}{2(K_{eq}-1)}; \text{(eq. 4)}$$

$$where\ K_{eq} \neq 1.$$

When the $K_{eq} = 1, \eta = \frac{\tau}{\gamma+\tau+1}$.

To quantitatively describe and compare sequence specificity, discrimination factor (DF) is commonly employed, where $DF = \eta_{correct}/\eta_{spurious}$. In a general case where neither correct ($K_{eq,c}$) nor spurious ($K_{eq,s}$) target has equilibrium constant as 1, the discrimination factor (DF) is expressed as:

$$DF = \frac{\eta_c}{\eta_s} = \frac{(K_{eq,s}-1)\cdot\left\{(K_{eq,c}+\gamma+K_{eq,c}\tau)-\sqrt[2]{(K_{eq,c}+\gamma+K_{eq,c}\tau)^2-4K_{eq,c}\tau(K_{eq,c}-1)}\right\}}{(K_{eq,c}-1)\cdot\left\{(K_{eq,s}+\gamma+K_{eq,s}\tau)-\sqrt[2]{(K_{eq,s}+\gamma+K_{eq,s}\tau)^2-4K_{eq,s}\tau(K_{eq,s}-1)}\right\}}; \text{ (eq. 5)}$$

For a well-designed probe where $K_{eq,c}$ is tuned to 1, the DF formula can be simplified as:

$$DF = \frac{\eta_c}{\eta_s} = \frac{2(K_{eq,s}-1)\cdot\tau}{(\gamma+\tau+1)\cdot\left\{(K_{eq,s}+\gamma+K_{eq,s}\tau)-\sqrt[2]{(K_{eq,s}+\gamma+K_{eq,s}\tau)^2-4K_{eq,s}\tau(K_{eq,s}-1)}\right\}}; \text{ (eq. 6)}$$

Practically, the DF value for a pair of correct and spurious targets is a function of both sequence design (ΔG and K_{eq}) and target concentration τ (eq. 5, 6).

The mathematical prediction of DF for a correct target against five spurious targets as a function of target concentration (τ) is shown in Supplementary Fig. 2a. DF values for all spurious targets containing single nucleotide mutations decrease monotonically with increasing target concentration τ. The simulated DF values maximize at the range of low target concentrations when the yield of the spurious target approaches extreme small values. However, when the concentration of the target is approach or below the limit of detection (LOD) of a specified analytical technique, the numeric values of yield and DF become meaningless. Despite the high DF, our simulation reveals that the absolute differences in yield between correct and spurious targets become much less at the concentration range $\tau < 0.6$ and are thus difficult to be resolved experimentally (Supplementary Fig. 2b). Therefore, we corrected our mathematical model by introducing the LOD of the analytical method into the simulation. The LOD can be defined arbitrary as a minimal yield that allows the experimental differentiation of signals generated by the correct or spurious targets from background. Supplementary Fig. 2c shows the simulation of DF using corrected model when the spurious becomes non-detectable (LOD was set to 1% yield). Practically, we can also set the LOD as the minimal detectable yield for the correct target.

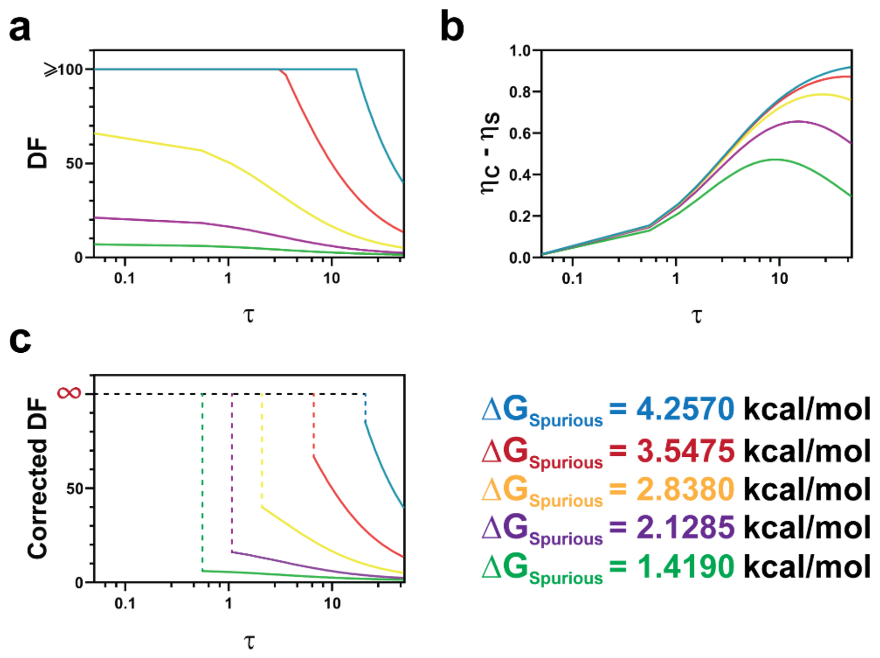

Supplementary Figure 2 | The theoretical concentration dependency of discrimination factor (DF). **a**. Theoretical DF as a function of target concentration for a correct target against five theoretical spurious targets defined by their thermodynamic parameters. Reaction free energies of spurious targets are shown with the same colors with DF curves. **b.** The differences in yield between a pair of correct and spurious targets as a function of target concentration. **c.** Simulation of DF by including the correction using LOD.

2.2 Robustness Factor

To quantitatively describe the detection window for discriminating SNVs, here we mathematically define a robustness factor (RF) which is the concentration ratio of a pair of spurious and correct targets, when their yields are the same. To do so, we first derived target concentration τ as a function of yield and equilibrium constant (eq. 7). RF can then be mathematically derived using eq. 8.

$$\tau = \frac{\eta \cdot (\gamma+\eta)}{K_{eq} \cdot (1-\eta)} + \eta; \text{ (eq. 7)}$$

$$RF = \frac{\tau_{spurious}}{\tau_{correct}} = \frac{\frac{\eta \cdot (\gamma+\eta)}{K_{eq,spurious} \cdot (1-\eta)}+\eta}{\frac{\eta \cdot (\gamma+\eta)}{K_{eq,correct} \cdot (1-\eta)}+\eta} = \frac{(1-\eta)+(\gamma+\eta) \cdot {}^{1}/_{K_{eq,spurious}}}{(1-\eta)+(\gamma+\eta) \cdot {}^{1}/_{K_{eq,correct}}}; \text{ (eq. 8)}$$

Under an optimal trade-off between sensitivity and specificity, where $K_{eq,correct} = 1$and the yield is 50%, a practically useful RF can be simplified as:

$$RF = \frac{0.5+(\gamma+0.5) \cdot {}^{1}/_{K_{eq,spurious}}}{0.5+(\gamma+0.5)}; \text{ (eq. 9)}$$

The theoretical RF values increase linearly as a function of η (Supplementary Fig. 3a). However, this is significantly deviated from the experimental observations, because the absolute concentration differences ($\tau_S - \tau_C$) between spurious and correct targets become much less significant, when the yields of hybridization approach 0 or 100% (Supplementary Fig. 3b). To better reflect the analytical performance, we corrected our model by taking both LOD and limit of linearity (LOL) into consideration. Supplementary Fig. 3e shows a corrected RF simulation by setting LOD to be 1% yield and LOL to 95% yield. To understand the concentration-dependency of RF, we further convert the x-axis from the yield η to the concentration of the target τ _correct (Supplementary Fig. 3c-e).

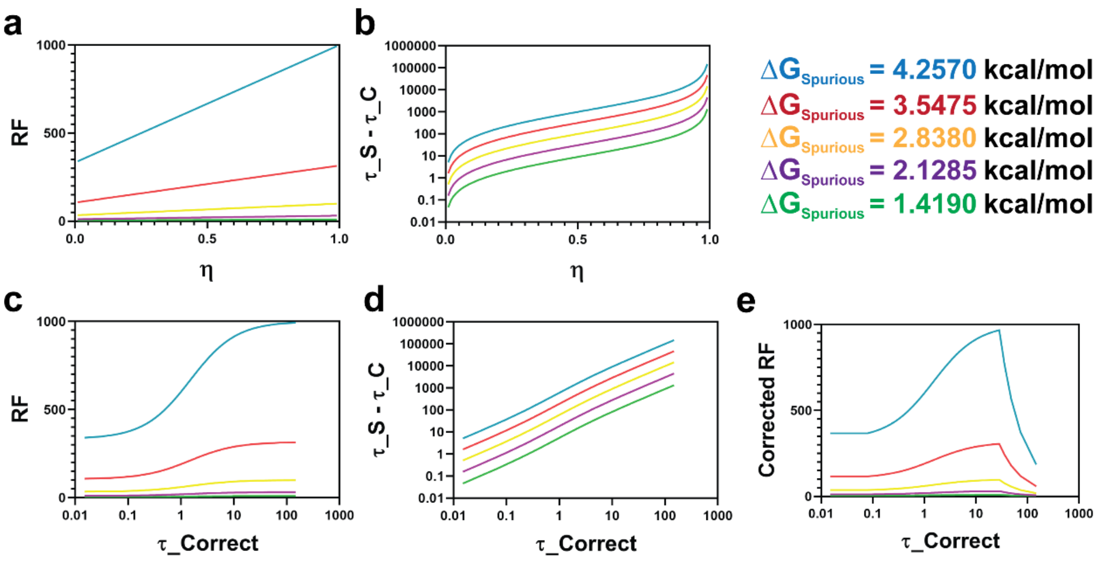

Supplementary Figure 3 | The dependence of RF on reaction yield and target concentration. a. The theoretical prediction of RF values as functions of reaction yield η. **b.** The absolute concentration differences between spurious and correct targets as a function of yield. **c.** RF as a function of target concentration. **d.** Absolute concentration differences at the same yield for the spurious and correct targets as a function of target concentration. **e.** Corrected RF using LOD and LOL.

2.3 Detection window of a toehold-exchange probe

To demonstrate the concentration-dependency of detection window for a toehold-exchange probe. We next simulated the η, DF, and RF using a numeric approach through MATLAB. A 42-nt synthetic DNA (see S5.1) was used as a model target and a single T to A mutation was introduced to create the spurious target. Standard Gibbs free energy (ΔG^0) of each DNA species can be calculated using NUPACK software and ΔG^0_{rxn} for each toehold-exchange reaction can thus be calculated as $\Delta G^0_{rxn} = \Delta G^0(TC) + \Delta G^0(P) - \Delta G^0(T) - \Delta G^0(CP)$. The thermodynamical differences between a pair of correct and spurious targets can be quantified using $\Delta\Delta G^0$, where $\Delta\Delta G^0 = \Delta G^0_{rxn}(spurious) - \Delta G^0_{rxn}(correct)$. In our model system, $\Delta\Delta G^0$ is determined to be at 2.30 kcal/mol. The yield (Supplementary Fig. 4a), sequence selectivity (Supplementary Fig. 4b), and concentration robustness (Supplementary Fig. 4c) can then be predicted for this pair of synthetic sequences in silico. As expected, all three parameters are strongly concentration-dependent (Supplementary Fig. 4a-4c), indicating that a well-designed and -optimized toehold-exchange probe may only excel in a certain concentration range.

By further including $\Delta\Delta G^0$ as a variable in our model, we were able to simulate the concentration-dependency of a toehold-exchange probe to all possible mutations that are mathematically reflected as varying $\Delta\Delta G^0$ values (Supplementary Fig. 4d-4f). Our simulation results quantitatively reflect that the detection window is inversely related to the difficulty for discriminating a certain mutation: the smaller the $\Delta\Delta G^0$ value, the narrower the concentration robustness range that allows effective discrimination (Supplementary Fig. 4e).

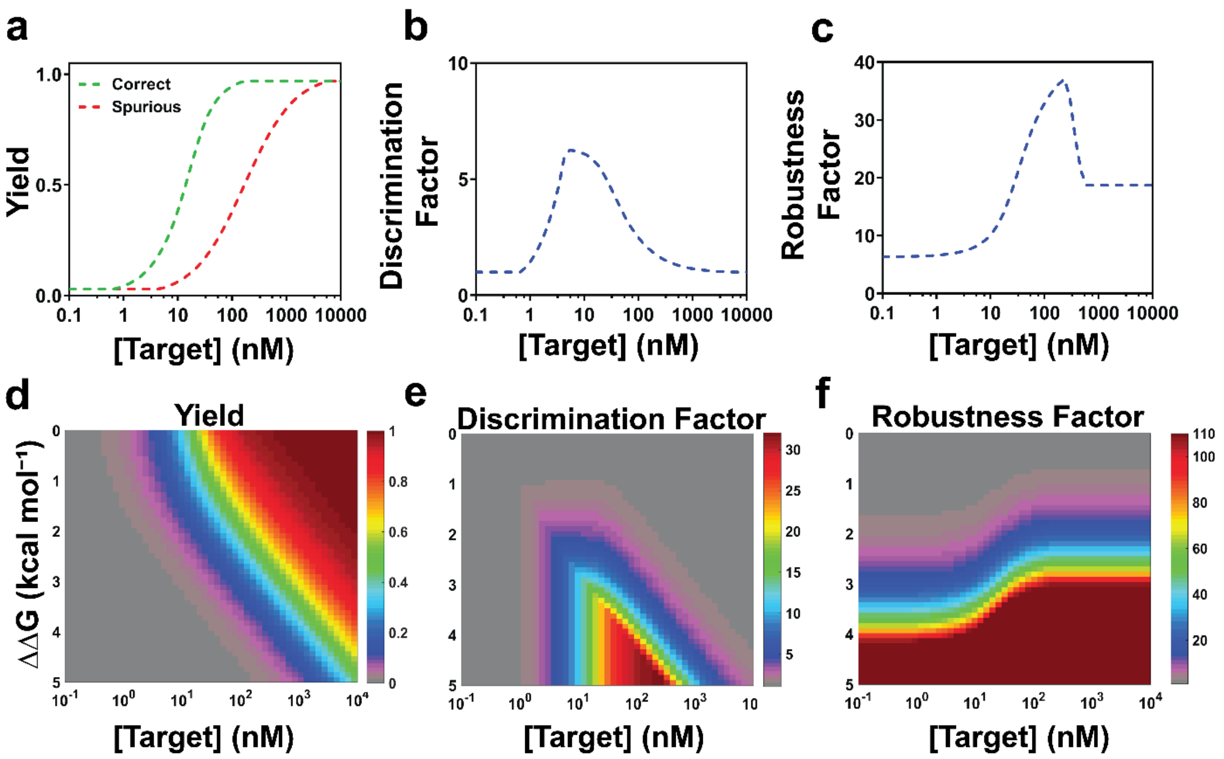

Supplementary Figure 4 | Detection window of nucleic acid hybridization probe. a-c. Theoretical prediction of the reaction yield, DF, RF of a pair of correct and spurious targets with $\Delta\Delta G^0 = 2.30\ kcal/mol$ through simulation. **d-f.** In silico profiling the yield, DF, and RF of all possible mutations with $\Delta\Delta G^0$ between 0 and 5 kcal/mol.

2.4 DNA Equalizer Gate

DEG is designed to convert a dsDNA target into a ssDNA output in a quantitative manner with well-defined detection window. To simulate this process, we consider that all reactions are thermodynamically driven, and all DNA species are in their thermodynamic stable states. Under this assumption, a set of equilibrium equations could be used to predict the concentration distribution of newly formed DNA species (Fig. 3a in the main content). However, only independent equations need to be solved otherwise meaningless answers will be generated. To help determine independent equilibrium equations, we extract a numerical reaction matrix (RM) from the reaction system:

$$RM = \begin{bmatrix} -1 & -1 & 1 & 0 & 0 & 0 & 0 & 0 \\ 0 & -1 & 0 & -1 & -1 & 1 & 0 & 0 \\ 0 & -1 & 0 & -1 & 0 & 0 & 1 & 0 \\ 0 & -1 & 0 & 0 & -1 & 0 & 0 & 1 \\ 0 & 0 & 0 & 0 & -1 & 1 & -1 & 0 \\ 0 & 0 & 0 & -1 & 0 & 1 & 0 & -1 \\ -1 & 0 & 1 & 1 & 0 & 0 & -1 & 0 \\ -1 & 0 & 1 & 0 & 1 & 0 & 0 & -1 \end{bmatrix};$$

In RM, each row represents a possible chemical reaction and Reactant is the column of all DNA species. The rank of RM is 4 (validated by Matlab), which is less than the dimension of RM. As such, only 4 independent equations are existing in this reaction system and we choose the first four reactions in our model. All ΔG^0_{rxn} values are predicted using NUPACK and the equilibrium equations are shown below:

$$\frac{[AB]_{eq}}{[A]_{eq}\cdot[B]_{eq}} = K_{eq,i} = e^{-\Delta G^0_i/RT}$$

$$\frac{[BCD]_{eq}}{[B]_{eq}\cdot[C]_{eq}\cdot[D]_{eq}} = K_{eq,ii} = e^{-\Delta G^0_{ii}/RT}$$

$$\frac{[BC]_{eq}}{[B]_{eq}\cdot[C]_{eq}} = K_{eq,iii} = e^{-\Delta G^0_{iii}/RT}$$

$$\frac{[BD]_{eq}}{[B]_{eq}\cdot[D]_{eq}} = K_{eq,iv} = e^{-\Delta G^0_{iv}/RT}$$

$$where\ [A]_0 = [A]_{eq} + [AB]_{eq};\ [B]_0 = [B]_{eq} + [BCD]_{eq} + [BC]_{eq} + [BD]_{eq};$$

$$[C]_0 = [C]_{eq} + [BC]_{eq};\ [D]_0 = [D]_{eq} + [BD]_{eq};$$

$$[A]_0, [B]_0, [C]_0, and\ [D]_0\ are\ initial\ concentrations;$$

Standard reaction free energies are calculated at 4 °C according to the experimental condition of DEG. When [Target] $>$ [DEP], a probability function is introduced to the model to quantitatively describe the probabilistic binding occurring between DEPs and complementary strand.

Supplementary Fig. 5 shows the mathematical prediction of the yield of each DNA species at varying target concentrations. The yield of the output DNA (A) decreases linearly as a function of the concentration of the input target without the correction using the probability function (dashed line in Supplementary Fig. 5a and 5b). With probability correction, a sharp transition occurs when [target] equals [DEP], which was also confirmed experimentally. This transition is determined exclusively by the concentration of DEP and thus allows defining the detection window in Supplementary Fig. 5c and suppressing the signals of spurious targets as shown in Supplementary Fig. 5d.

The combination of DEG model with a classic toehold-exchange model allows us to precisely simulate the yield and discrimination factor for the correct target and any given mutation. To simulate RF in DEG system, a build-in mathematical reverse function in Matlab was used to first convert the reaction yield to the concentration of the ssDNA output using the toehold-exchange model and then convert the concentration of ssDNA to that of the dsDNA target using the DEG model.

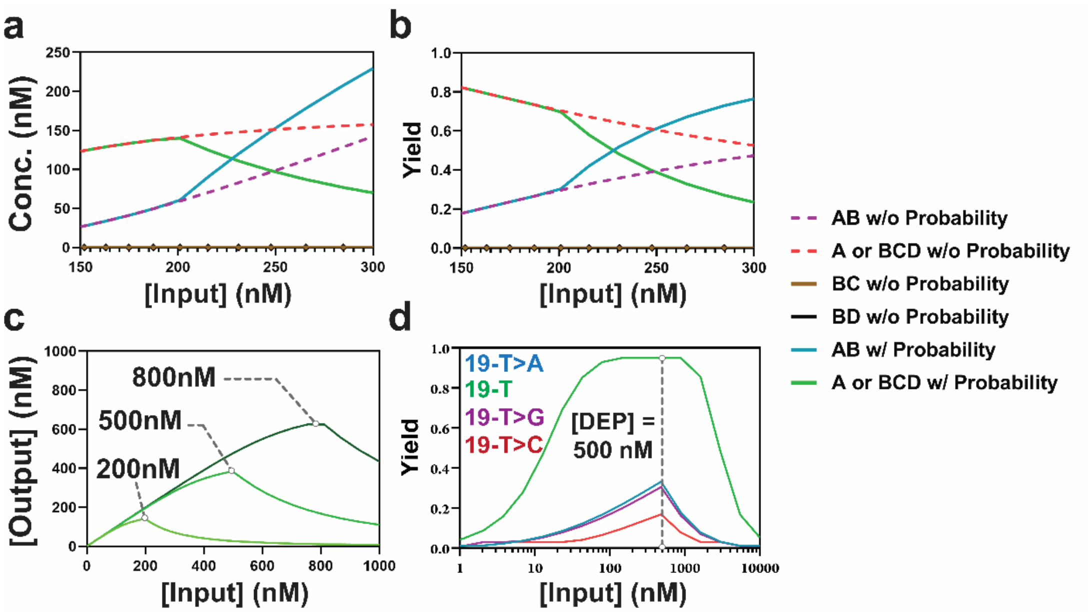

Supplementary Figure 5 | Theoretical predictions of yields for producing ssDNA output A from dsDNA input AB using DEG. a. The output ssDNA (A) and input dsDNA (AB) concentrations as a function of the initial input concentration. The probability function plays critical roles to ensure the accuracy of the model. **b.** The yield of ssDNA (A) as a function of the initial input concentration. **c.** Effect of DEPs on the performance of DEG for producing ssDNA output. A maximum yield exists for each titration curve, where [Input] = [DEP]. **d.** Titration curve for analyzing a correct dsDNA target against three single nucleotide mutations. [DEP] = 500 nM.

2. 5 Comparison between DEG and increase in energy barrier for expanding the detection window

An already established detection window for discriminating a single nucleotide mismatch (left in Supplementary Fig. 6) can be enlarged either through increase in energy barriers for activating the probe (right, bottom in Supplementary Fig. 6) or using our DEG approach (right, up in Supplementary Fig. 6). As demonstrated by the simulation results in Supplementary Fig. 6, our DEG approach works better in both the degree of expansion (essentially to infinite) and more sensitive at low concentration range.

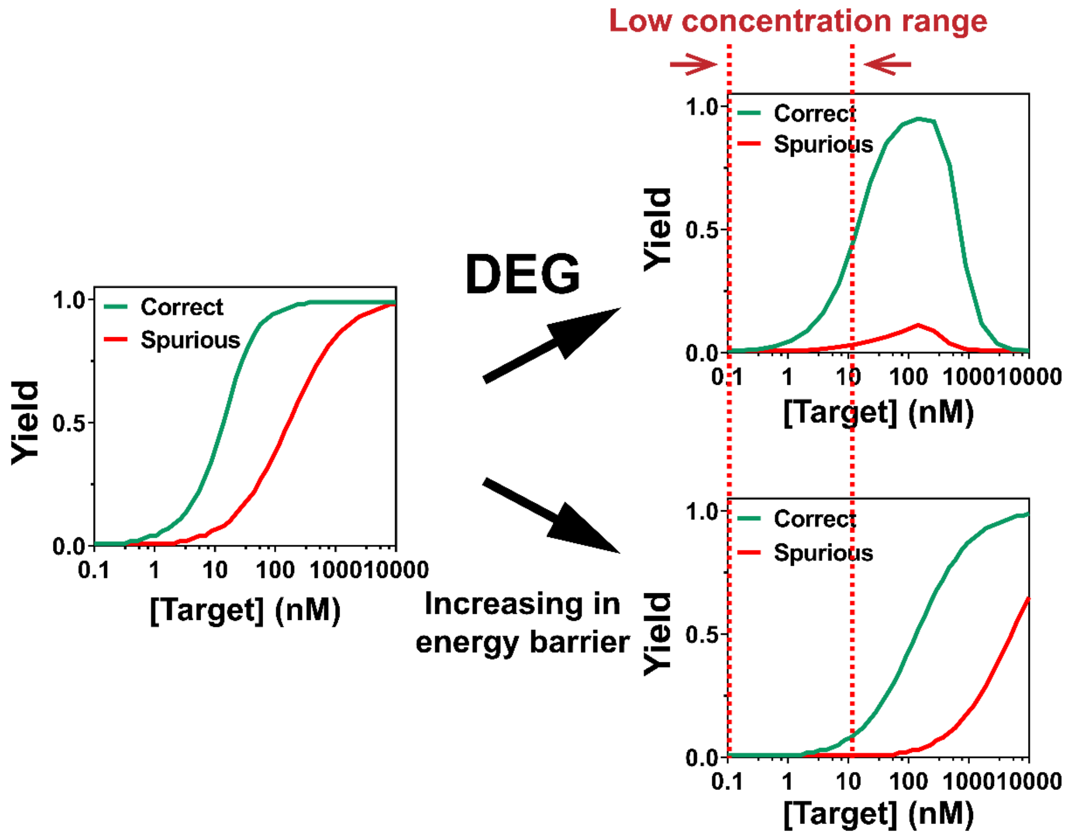

Supplementary Figure 6 | Simulation results of enlarged detection windows achieved through DEG (Top) and the increase in the energy barrier for activating the toehold-exchange probe (Bottom). Increase in the energy barrier is achieved by elongating the reverse toehold by 2 bp.

2.6 Parameter correction and fitting

2.6.1 Correction of ΔG^0_{rxn}

It was previously found by Zhang and colleagues that corrections to ΔG^0_{rxn} values predicted using NUPACK software are necessary to improve the agreement between theoretical prediction and experimental observation.[1] A similar correction was also performed in our study to improve the accuracy of the mathematical prediction (Supplementary Fig. 7). By comparing theoretically predicted and experimentally determined yields at varying ΔG^0_{rxn}, a correction of 1.575 kcal/mol was determined and applied throughout this study.

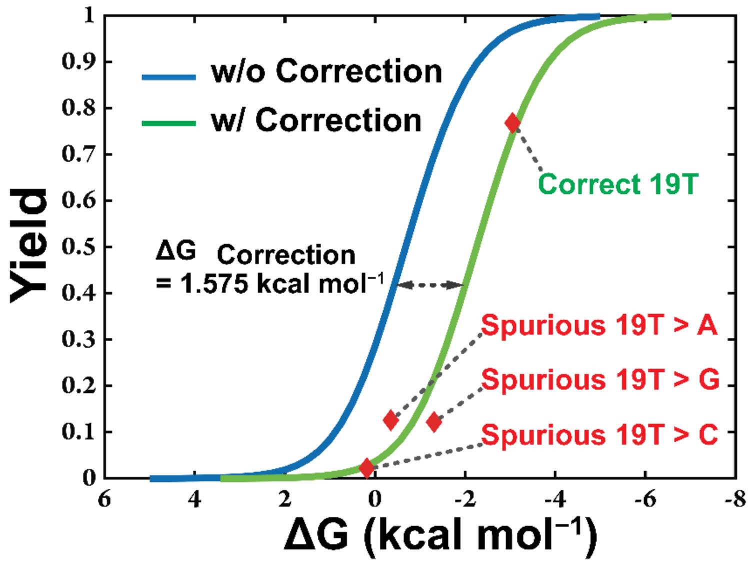

Supplementary Figure 7 | Correction of ΔG^0_{rxn}. The correction was performed by measuring a correct target and three single nucleotide mutations using a toehold-exchange reporter. The yield can be predicted using predicted ΔG for each DNA species (blue curve). To fit the theoretical curve with experimental results, a correction of -1.575 kcal/mol was found to be necessary. This correction was applied to all simulations throughout this work. [AB] = 10 nM, [reporter] = 20 nM, theoretical ΔG for all DNA species were predicted using NUPACK software.

2.6.2 Determination of experimental RF through fitting

As both calibration curves for the correct and spurious targets were established using scattered data spots, it is not possible to determine the experimental RF directly. Therefore, we combined experimental fitting and mathematical conversion to address this issue (Supplementary Fig. 8). A 4-parameter nonlinear fitting was used first to fit the experimental results. A set of four parameters including M, L, s and E, will be determined through the fitting (eq. 12). M and L represents the highest and lowest signals in the curve; E represents the concentration of target that gives halfway between maximum and minimum limits; and $\boldsymbol{s}$ represents the steepness of the fitting curve. Once established this mathematical model through fitting, we were able to convert any yield in a toehold-exchange reaction into a corresponding concentration of either a correct or spurious target. Experimental RF can then be determined. For a DEG system, a calibration curve needs to be first split into two parts: $[Target] =< [DEP]$ and $[Target] > [DEP]$ (Supplementary Fig. 8).

Nonlinear Model:

$$Y = \frac{L + x^S \cdot (M - L)}{x^S + E^S} \text{ (eq. 10)}$$

$$where\ M, L, s\ and\ E\ are\ parameters\ to\ be\ fitted.$$

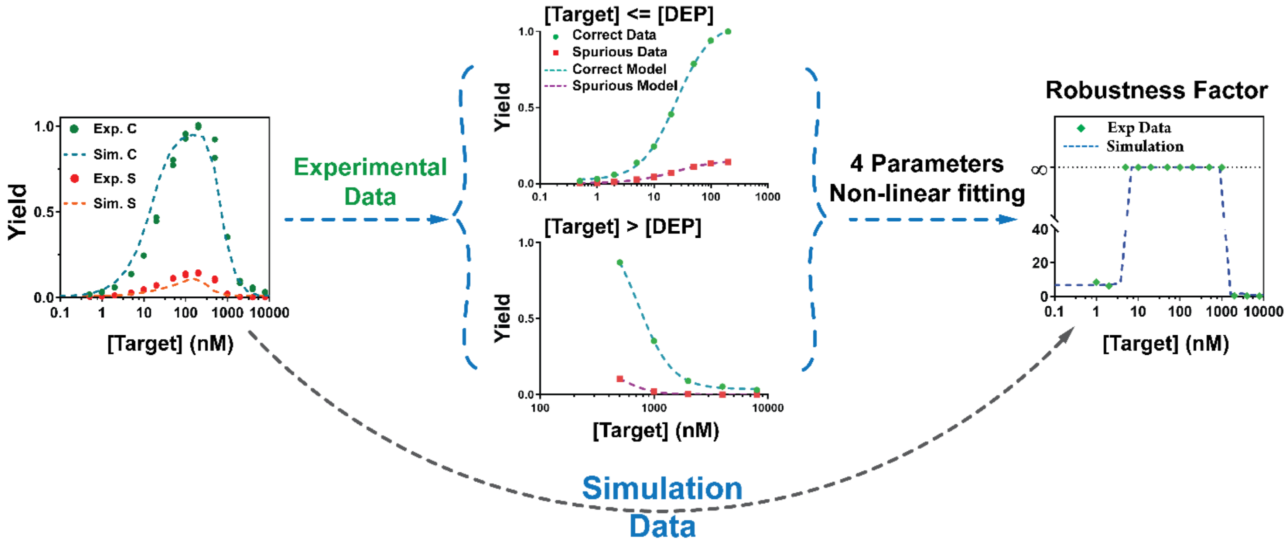

Supplementary Figure 8 | Determination of theoretical and experimental RF. The theoretical RF is determined by extracting the concentrations of a pair of correct and spurious targets producing the same yield using Matlab software. The experimental RF is determined by first fitting experimental data in a calibration curve using a 4-parameter non-linear model and then extracting the concentrations of a pair of correct and spurious targets using Matlab. RF at each concentration is then calculated using eq. 6 and plotted as a function of target (correct target) concentrations. Experimentally measured yields are shown in empty dots (n=2).

3. Experimental validation and optimization of DEG

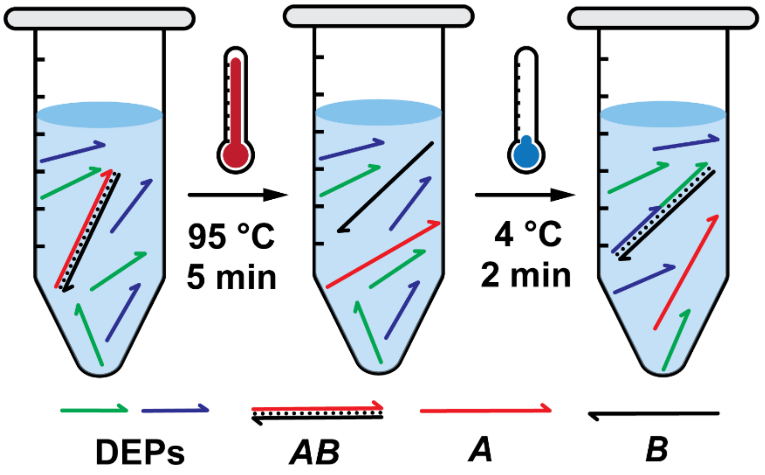

Supplementary Figure 9 | Scheme illustration of the experimental procedures and DNA reactions in DEG. Double-stranded DNA (dsDNA) target AB is mixed with DEPs at a proper ratio. The solution was then heated to 95 °C, where AB is denatured into A and B. The reaction is then quenched using a snap cooling protocol that cools the reaction to 4 °C within 2 min. DEPs then competitively bind to B, which generates A as the output for the subsequent quantitative analysis.

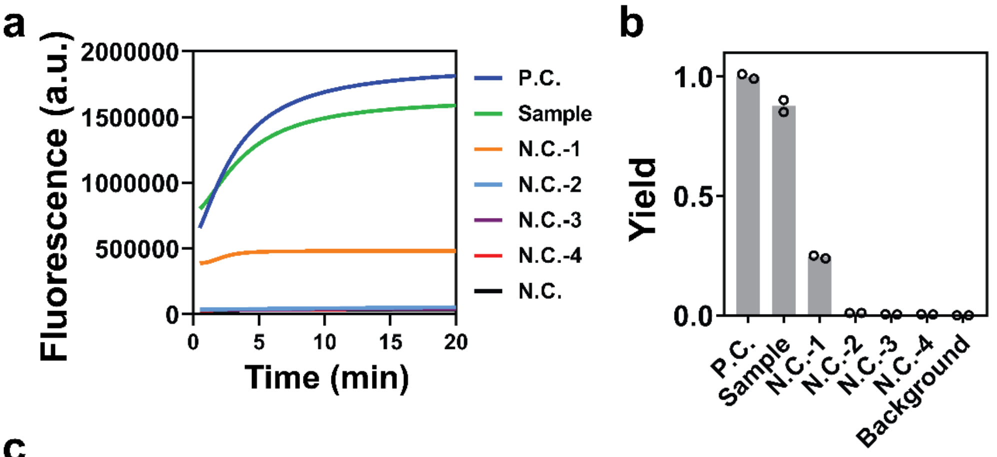

c

	Target	DEPs	Thermal Protocol	Exchange Reporter
P. C.	ssDNA			✓
Sample	dsDNA	✓	✓	✓
N.C. 1	dsDNA	✗	✓	✓
N.C. 2	dsDNA	✓	✗	✓
N.C. 3	—	✓	✓	✓
N.C. 4	—	✓	✗	✓
N.C.	—			✓

Supplementary Figure 10 | Characterization the effect of DEPs and thermal protocols on the performance of DEG. Both DEPs and thermal protocols are critical to ensure the high yield of DEG for producing single-stranded output A. **a.** Real-time fluorescence monitoring the kinetics of the reporter probe for measuring A produced by DEG. **b.** Yield of each reaction in terms of producing A measured by endpoint fluorescence at 20 min. The yield was calculated by setting fluorescence of the positive control as 1. Bars represent the mean of individual replicates (circles, n=2). **c.** Detailed reactants and experimental procedures in each sample or control. Each sample containing 10 nM AB, 20 nM reporter, and 200 nM DEPs in 1 × Tris buffer containing 1 mM Mg^{2+} and 0.1% of Tween 20 (v/v) was incubated at 37 °C.

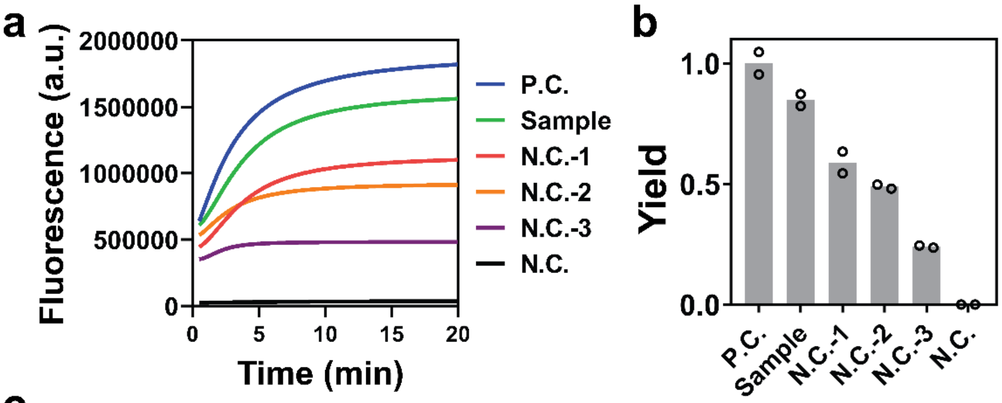

c

	Target	DEP-1	DEP-2	Exchange Reporter
P. C.	ssDNA			✓
Sample	dsDNA	✓	✓	✓
N.C. 1	dsDNA	✓	✗	✓
N.C. 2	dsDNA	✗	✓	✓
N.C. 3	dsDNA	✗	✗	✓
N.C.	——			✓

Supplementary Figure 11 | Effect of DEPs on the performance of DEG. Each DEP probe was found to partially complement with B and thus facilitate the conversion of AB to A. However, maximum yield can only be achieved when both DEPs are present in the reaction. **a.** Real-time fluorescence monitoring the kinetics of the reporter probe for measuring A produced by DEG. **b.** Yield of each reaction in terms of producing A measured by endpoint fluorescence at 20 min. Bars represent the mean of individual replicates (circles, n=2). **c.** Detailed reactants and experimental procedures in each sample or control.

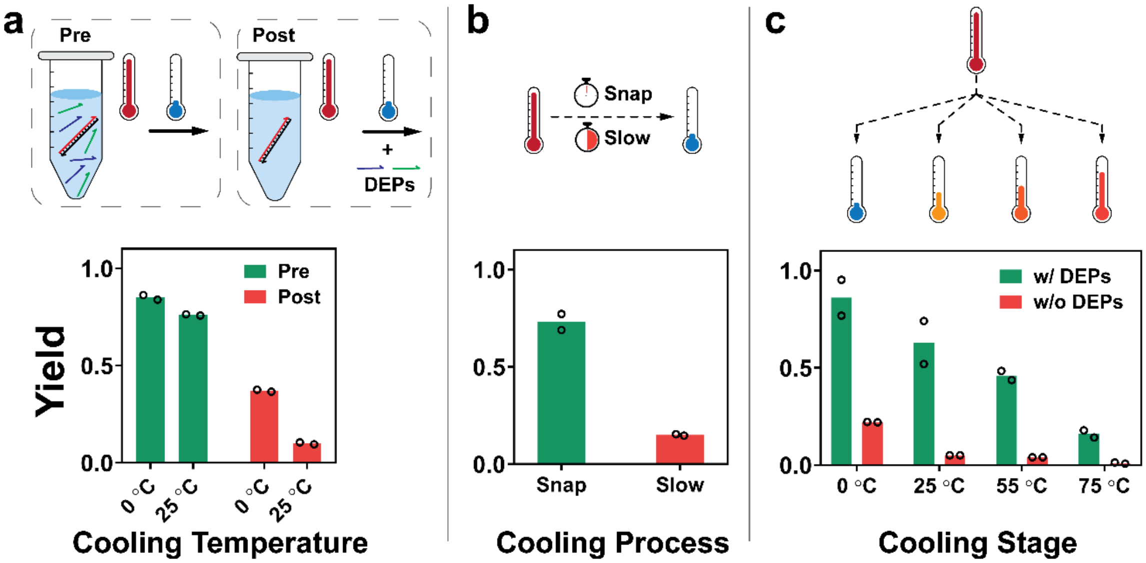

Supplementary Figure 12 | Optimization of the thermal protocol for the denaturation and renaturation processes. Maximal yield is established when all DNA species pre-mixed in the same test tube and undergo a heating followed by a snap cooling step to 4 °C. **a.** The addition of DEPs before (pre) or after (post) the thermal protocol was found to significantly affect the yield of A. A premixing of DEPs and the target was chosen as the optimal procedure, as it both improves the reaction yield and simplifies the operation **b.** A snap cooling step was also found to be critical to ensure the high yield of A. **c.** Maximal yield was achieved when a snap cooling to 4 °C was used as a final temperature. Increase final temperature to 25, 55 and 75 °C (blue, yellow, orange and red thermometer, respectively) were found to gradually reduce the yield of the reaction. Bars represent the mean of individual replicates (circles, n=2).

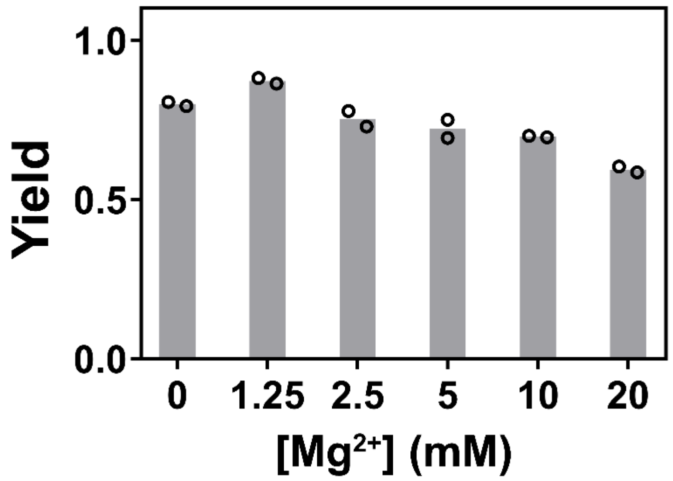

Supplementary Figure 13 | Effect of Mg^{2+} on the performance of DEG. The DEG was found to be robust to Mg^{2+} in range of 0 to 10 mM. A slight decrease in reaction yield was found when increasing Mg^{2+} concentration to 20 mM, as high concentration of Mg^{2+} may favor the formation of AB by accelerating renaturation. Bars represent the mean of individual replicates (circles, n=2).

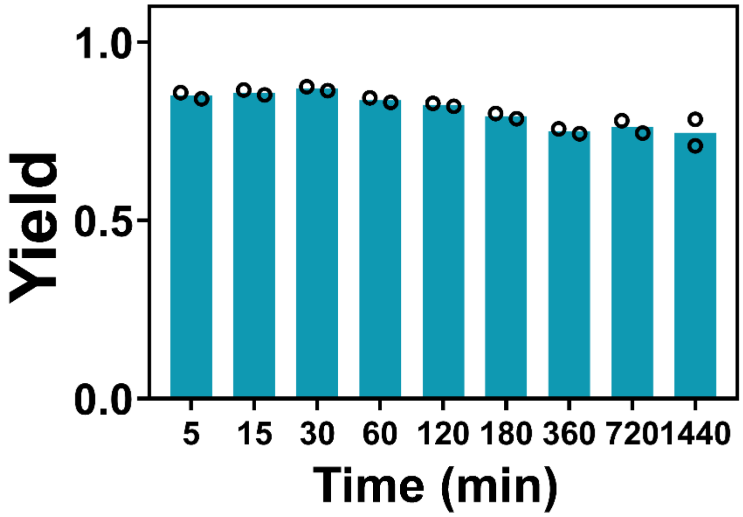

Supplementary Figure 14 | Stability of the output ssDNA *A* produced by DEG. Once generated by DEG, B is blocked by DEPs and thus unable to react with A through renaturation. To accurately quantify AB through DEG and the reporter, it is critical to ensure the stability of free A in the reaction mixture. We monitored the concentration of A in the solution after DEG reaction at 5 min, 15min, 30 min, 1 hr, 2 hr, 3 hr, 6 hr, 12 hr, and 24 hr, respectively, at room temperature. Our result demonstrates that A is highly stable, with no apparent losses in the first 2 hrs. Practically, we analyze A using the reporter probe within the first 30 min. Bars represent the mean of individual replicates (circles, n=2).

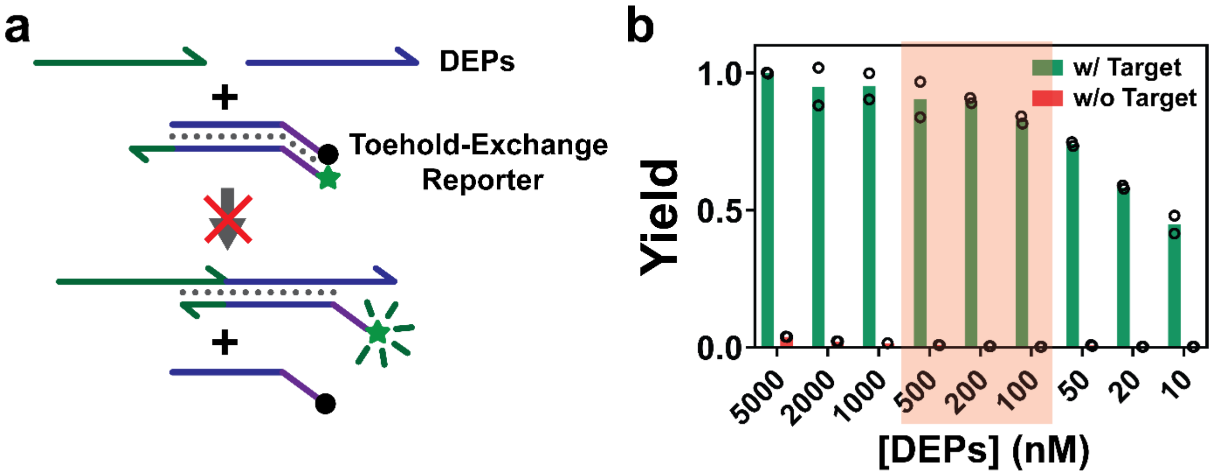

Supplementary Figure 15 | Estimation of signal leakage from DEPs. A possible source of fluorescence background is the signal caused by the interaction of DEPs and the reporter probe. Therefore, we estimated the signal leakage at varying DEP concentrations. **a.** Schematic illustration of the signal leakage caused by the interaction among DEPs and the reporter. **b.** Estimated leakage (w/o Target) as a function of DEP concentrations, which is also compared to the target-specific fluorescence (w/ Target). The target concentration is fixed at 10 nM, concentrations of DEPs are varied from 10 nM to 5 μM. No apparent fluorescence signal was observed when treating the fluorescence probe with up to 1 μM DEPs, suggesting that there was no cross-reaction between DEPs and the probe and thus there was no competition between DEPs and ssDNA output for the probe. Bars represent the mean of individual replicates (circles, n=2).

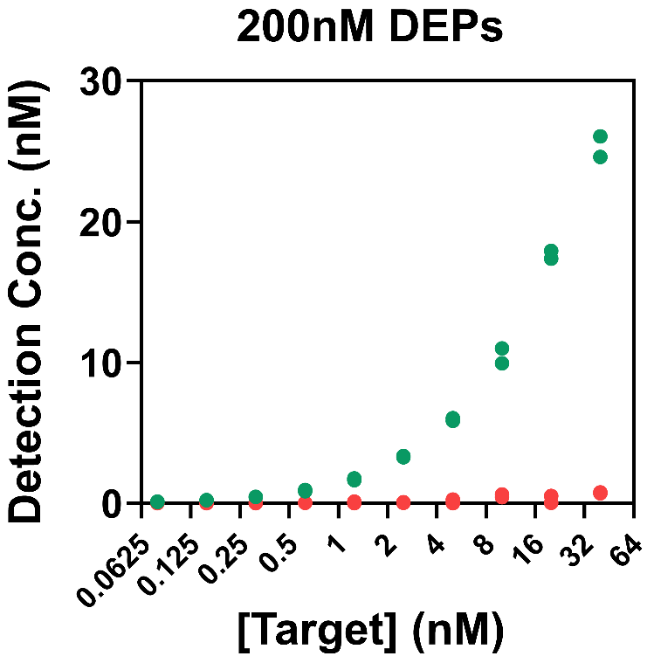

Supplementary Figure 16 | Estimated the LOD for detecting *AB* using DEG. The LOD was estimated to be 0.5 nM when 200 nM DEPs were used to generate single-stranded output A. Values of individual replicates (n=2) are shown as dots.

4. Length effect of target and DEP

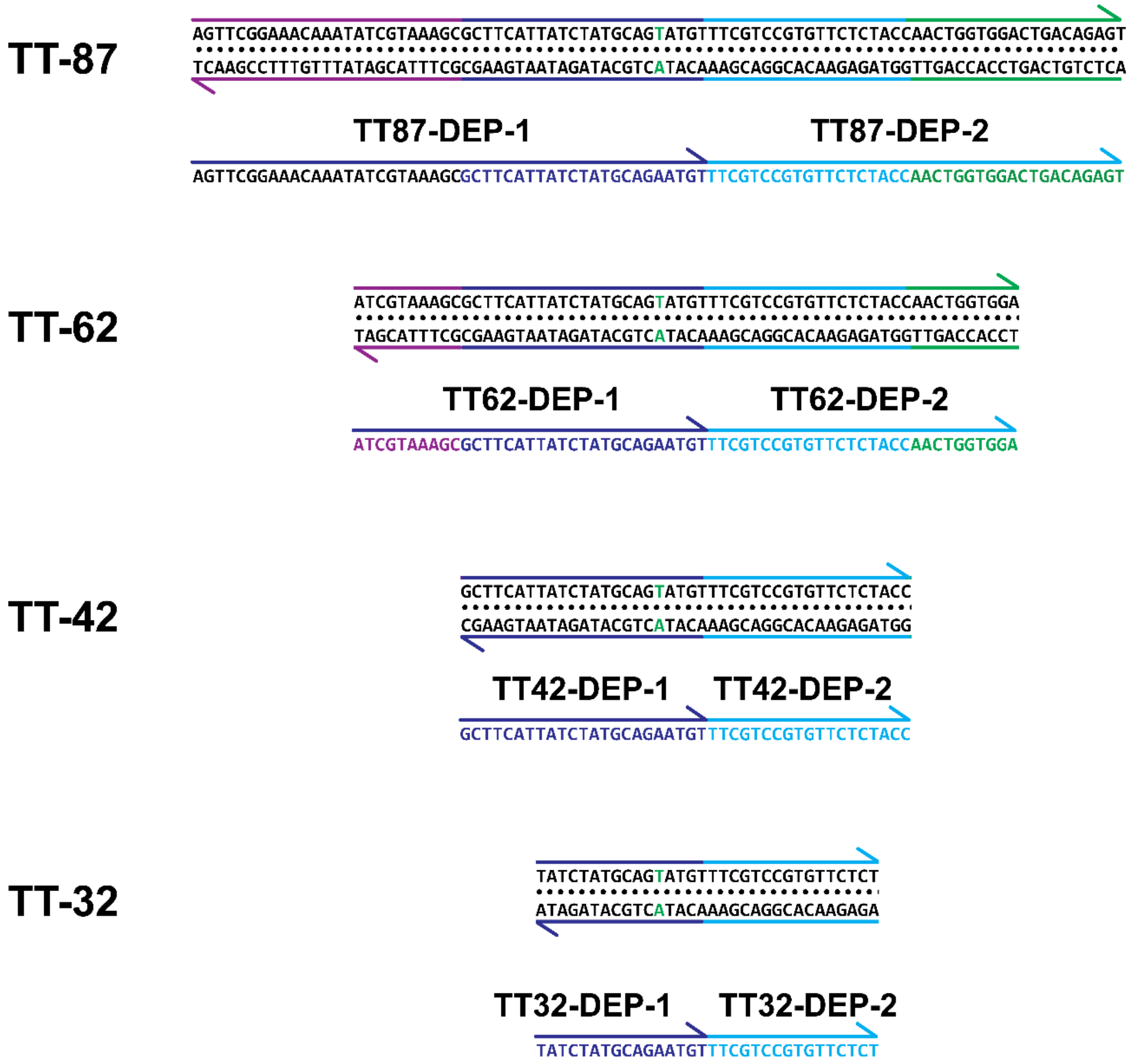

Supplementary Figure 17 | Sequence for varying length of TT targets and corresponding DEPs to validate the length effect of target/DEPs.

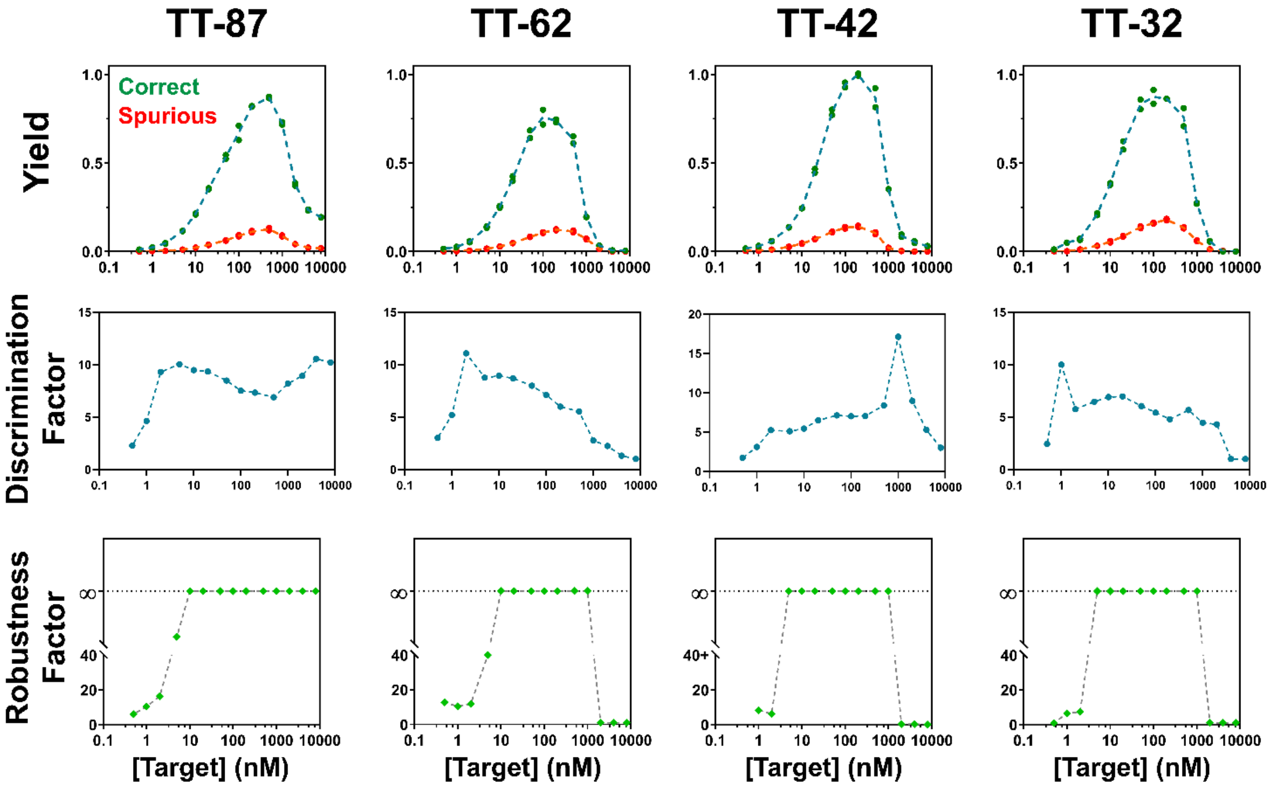

Supplementary Figure 18 | Experimentally measured yields, DFs, and RFs for varying length of dsDNA targets from 87 bp to 32 bp using corresponding DEPs at concentrations of 200 nM. These results suggest that our DEG approach is workable for targets with varying length ranges with minimal impact to the analytical performance. Individual replicates (n=2) of yield are shown as dots, and lines represent the mean values (correct and spurious yield curves).

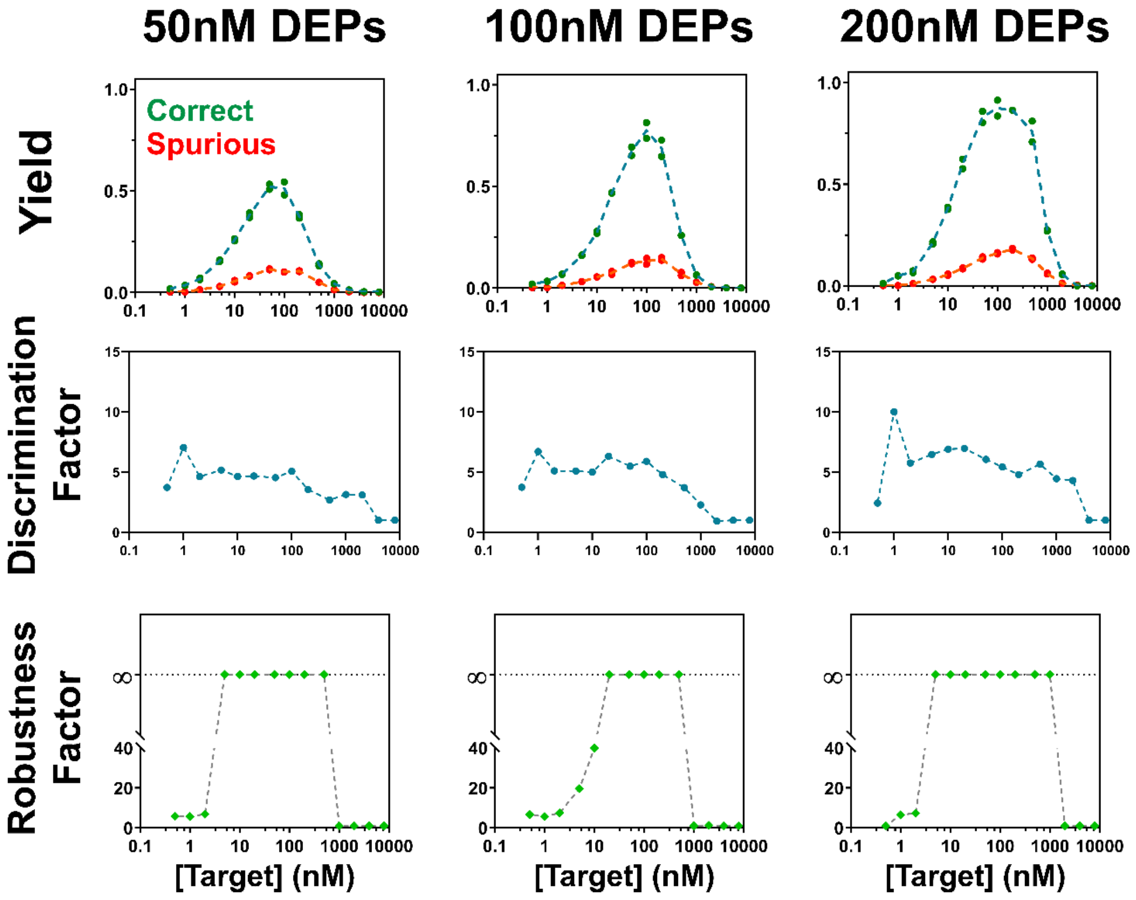

Supplementary Figure 19 | Experimentally measured yields, DFs, and RFs for target TT-32 using DEPs at concentration of 50, 100, and 200 nM, respectively. Individual replicates (n=2) of yield are shown as dots, and lines represent the mean values (correct and spurious yield curves).

5. Detection of varying single nucleotide mutations using DEG

We examined the analytical performance and versatility of DEG for discriminating single nucleotide variants using two sets of synthetic targets: a set of subgenomes (28 to 87 bp) from a β-tubulin gene of a parasitic worm, *Trichuris trichiura* (TT) and a 44 bp subgenomic sequence from *Hepatitis B Virus* (HBV) S gene. Both diseases are major threat to human health worldwide. Varying types of mutations and indels were tested using our DEG detection platform. Various synthetic cancer targets carrying single-nucleotide-mutation hot spots were used to represent the robustness and clinical application potential of our DEG method. We also demonstrated the possibility of multiplexed DEG by mixing the two sets of targets and corresponding DEPs into the same test tube.

5.1 Detection of single nucleotide mutations in *Trichuris trichiura*

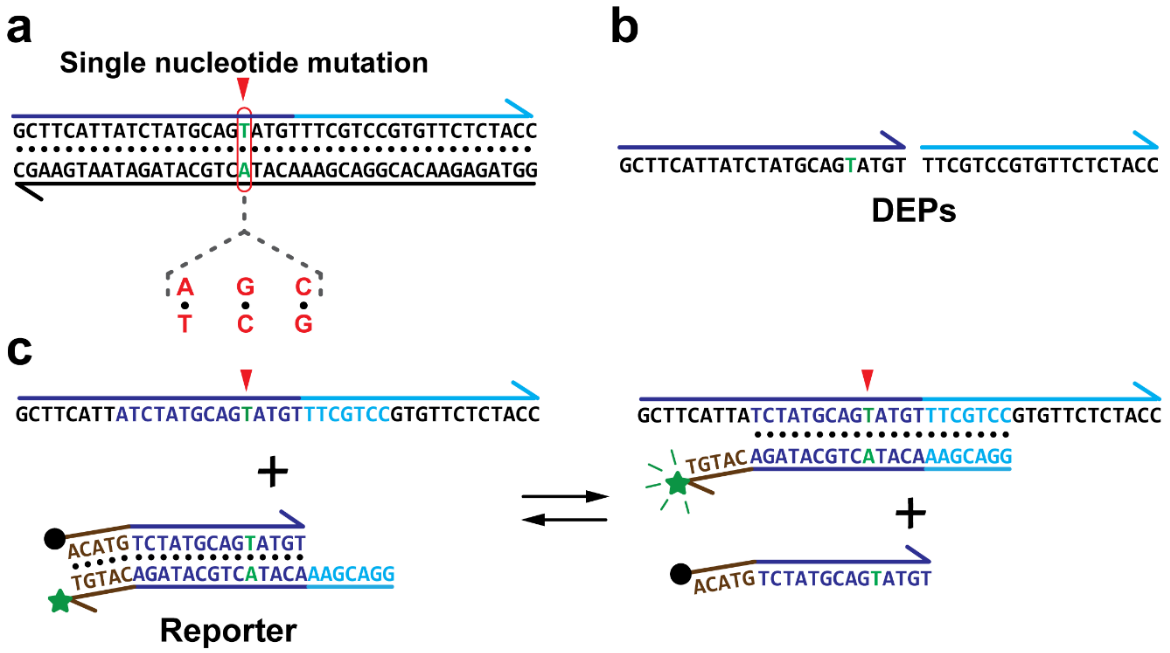

Supplementary Figure 20 | Schematic illustration of analyzing single nucleotide mutations of a subgenome of *Trichuris trichiura* (TT). a. Sequences and point mutations of the TT target. **b.** Sequences

of a pair of DEPs designed for the TT target. **c.** The sequence design of the reporter probe operated by the toehold-exchange.

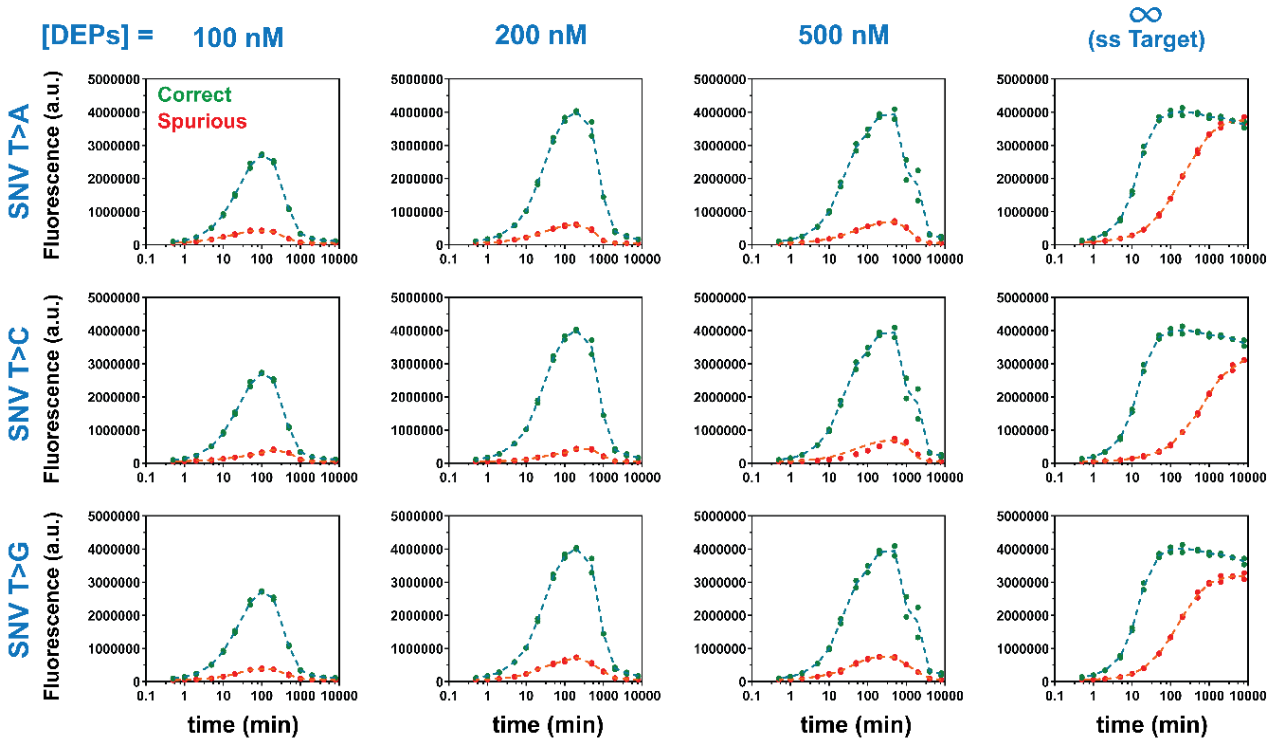

Supplementary Figure 21 | Experimental validation of DEG for discriminating single nucleotide T > A, T > G, and T > C mutations in the 42-bp dsDNA TT target. Raw fluorescence signals for detecting correct TT target and three single nucleotide mutations at DEP concentrations from 100 nM, to 200 nM, and to 500 nM. Single-stranded TT target was also analyzed directly using the reporter probe, which equivalent to a DEG system with infinite DEPs. Individual replicates (n=2) of yield are shown as dots, and lines represent the mean values (correct and spurious yield curves).

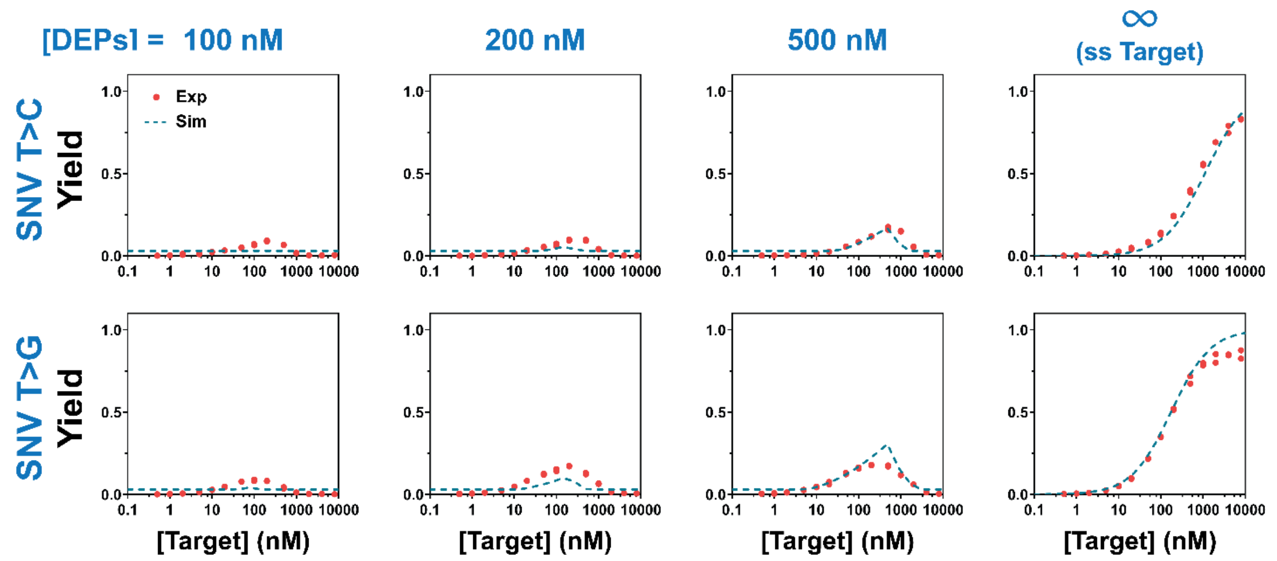

Supplementary Figure 22 | Comparison of experimentally determined yields with those predicted through simulation for analyzing T>G and T>C mutations to the double-stranded TT targets using DEG. Values of individual replicates (n=2) are shown as dots, and lines represent the simulation prediction.

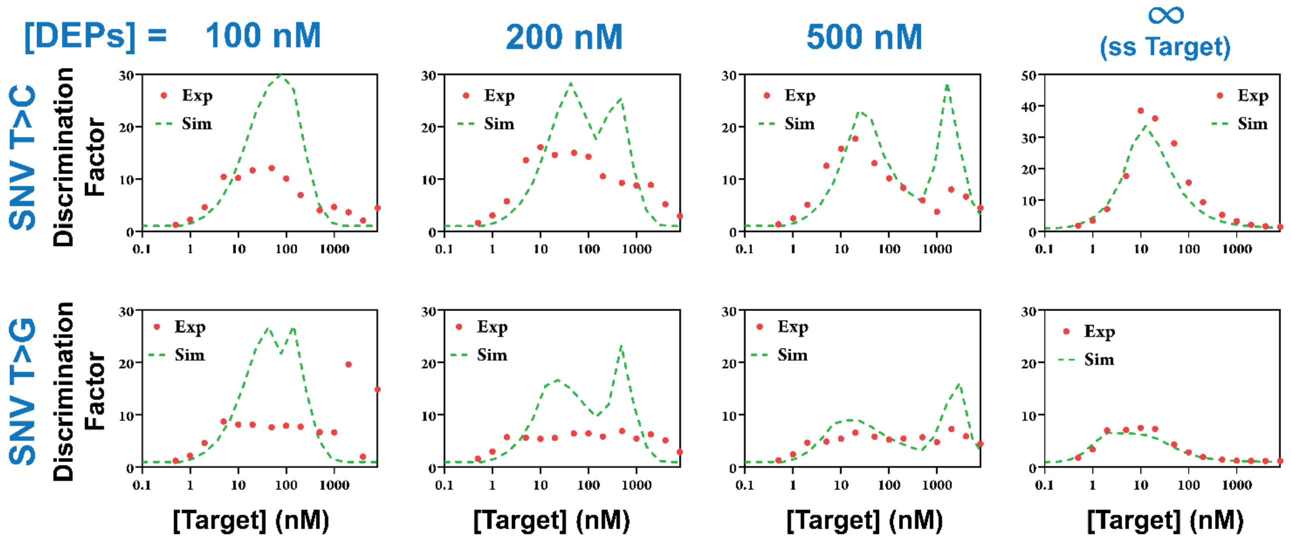

Supplementary Figure 23 | Comparison of experimentally measured and simulated DF for T > G and T > C mutations in the double-stranded TT target using DEG. Experimentally measured DF values are shown as dots, and lines represent the simulation prediction.

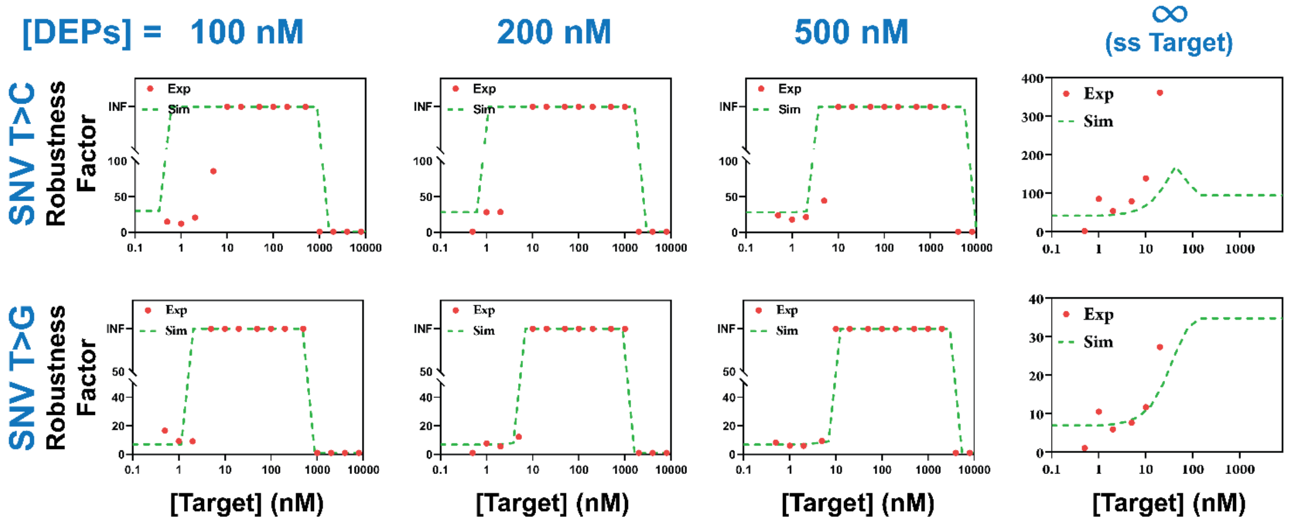

Supplementary Figure 24 | Comparison of experimentally measured and simulated RF for T > G and T > C mutations in the double-stranded TT target using DEG. Experimentally measured RF values are shown as dots, and lines represent the simulation prediction.

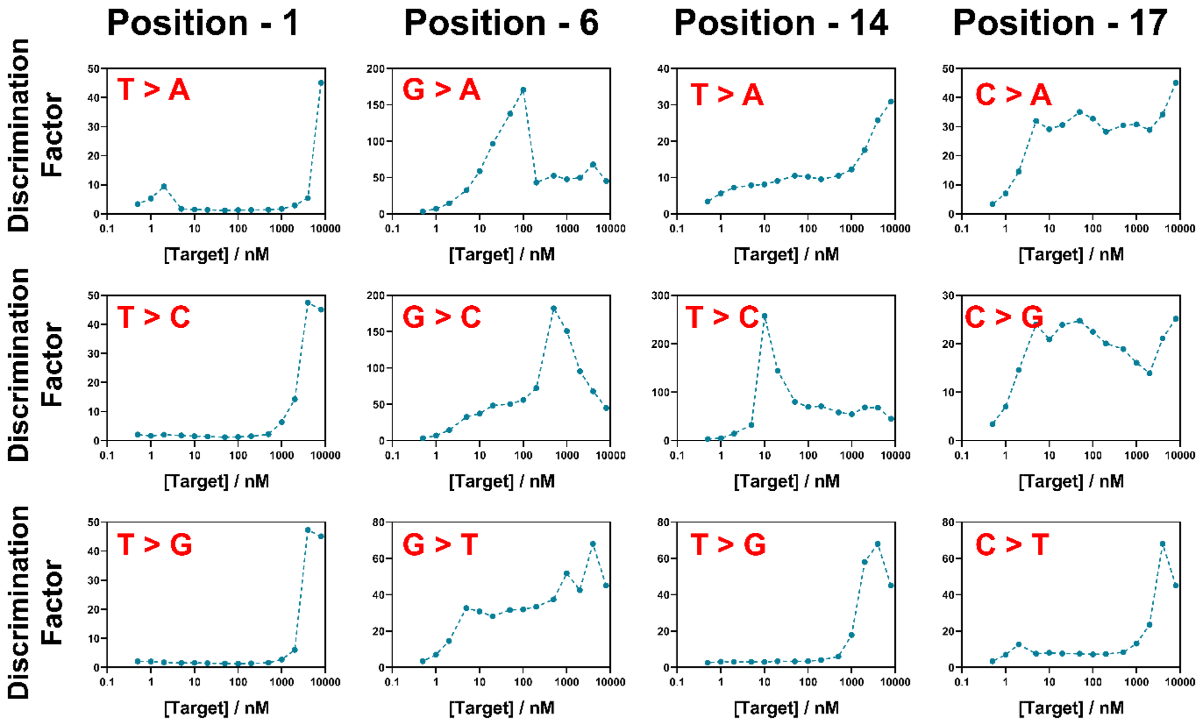

Supplementary Figure 25 | Experimentally measured DF of target TT-28 with different mutations.

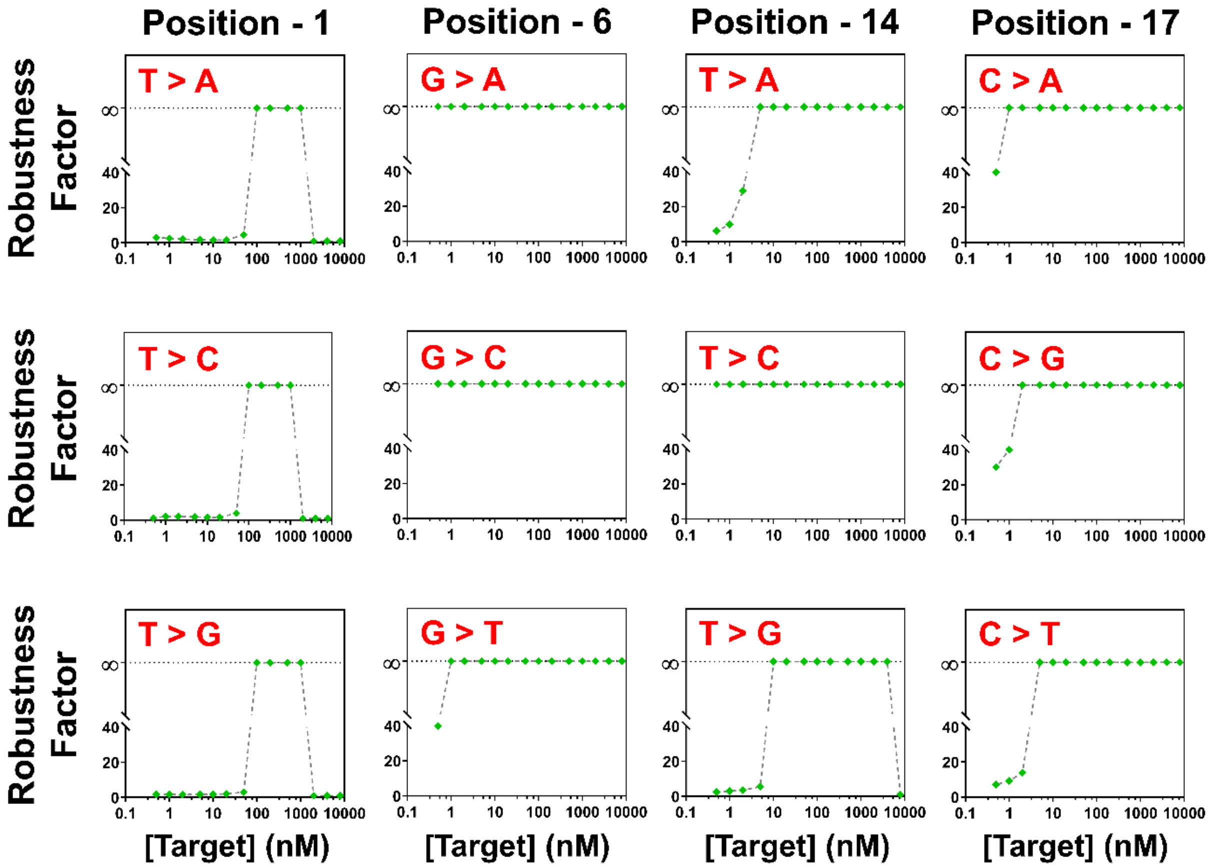

Supplementary Figure 26 | Experimentally measured RF of target TT-28 with different mutations.

5.2 Detection of single nucleotide mutations in HBV

The goal of designing the double-stranded synthetic HBV S-gene target is to test the versatility of our DEG approach. As shown in Supplementary Fig. 27, a pair of DEPs and a reporter probe were designed for this synthetic target. Both single nucleotide mutations and base insertion/deletion were introduced and tested in this system using DEG. To verify the DEG approach for discriminating challenging single nucleotide mutations, we intentionally introduced an A to G mutation, a well-known challenging SNV because of the formation of G-T wobble that reduces differences in free energies between correct and spurious targets. We found that our DEG approach has effectively improved the specificity and concentration robustness for analyzing this challenging SNV comparing to the direct analysis using the toehold-exchange beacon (highlights in Supplementary Fig. 29 and 30).

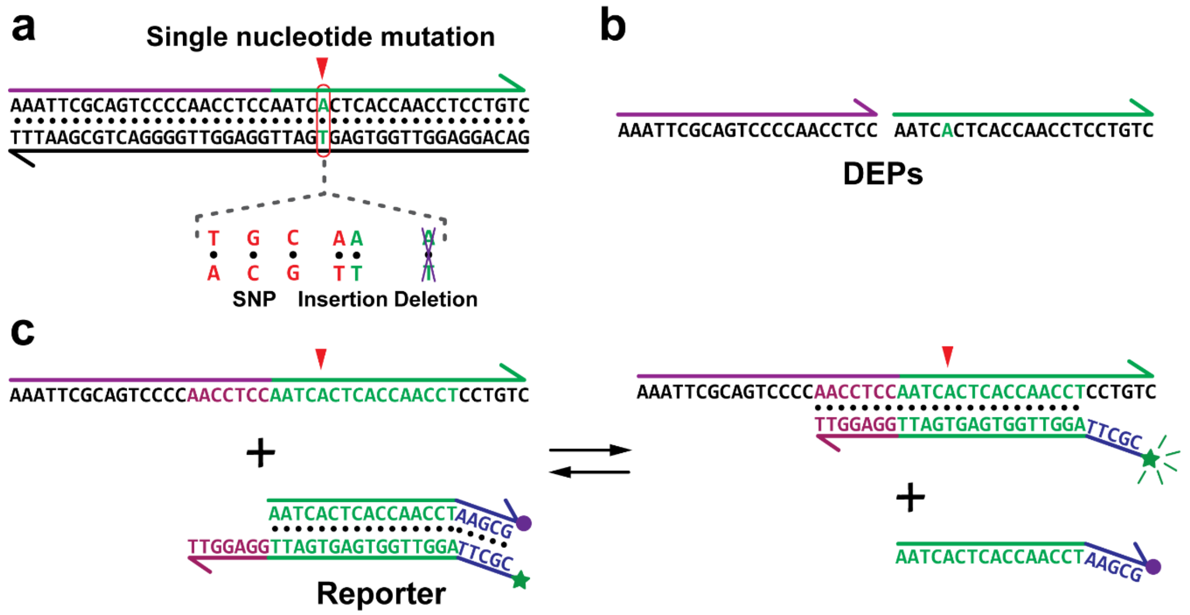

Supplementary Figure 27 | Schematic illustration of analyzing single nucleotide mutations of a subgenome of *HBV S gene*. a. Sequences and point mutations of the target. **b.** Sequences of a pair of DEPs designed for the HBV target. **c.** The design of the reporter probe operated by the toehold-exchange.

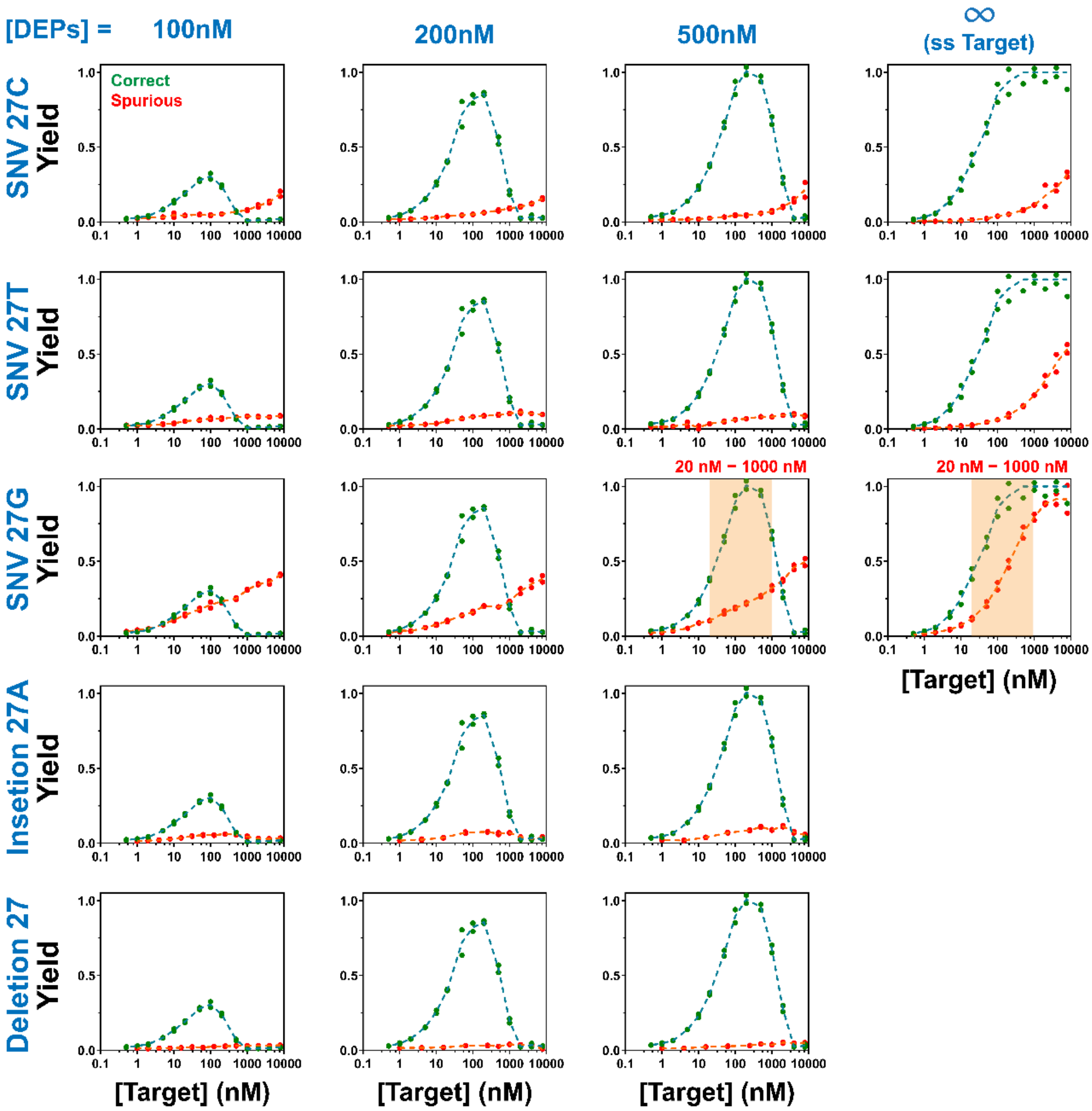

Supplementary Figure 28 | Detection of the synthetic HBV target with varying mutations and indels using DEG. Five SNVs, including three single nucleotide mutations, one insertion (INS) and one deletion (DEL), were tested using DEG with varying DEP concentrations from 100 nM, to 200 nM, and to 500 nM. Detection single nucleotide mutations in single-stranded HBV target was also included for mutations at 27C, 27T, and 27G. Shaded area indicates the detection window where DEG outperforms toehold-exchange beacon in discriminating the most challenge SNV27G. Individual replicates (n=2) of yield are shown as dots, and lines represent the mean values.

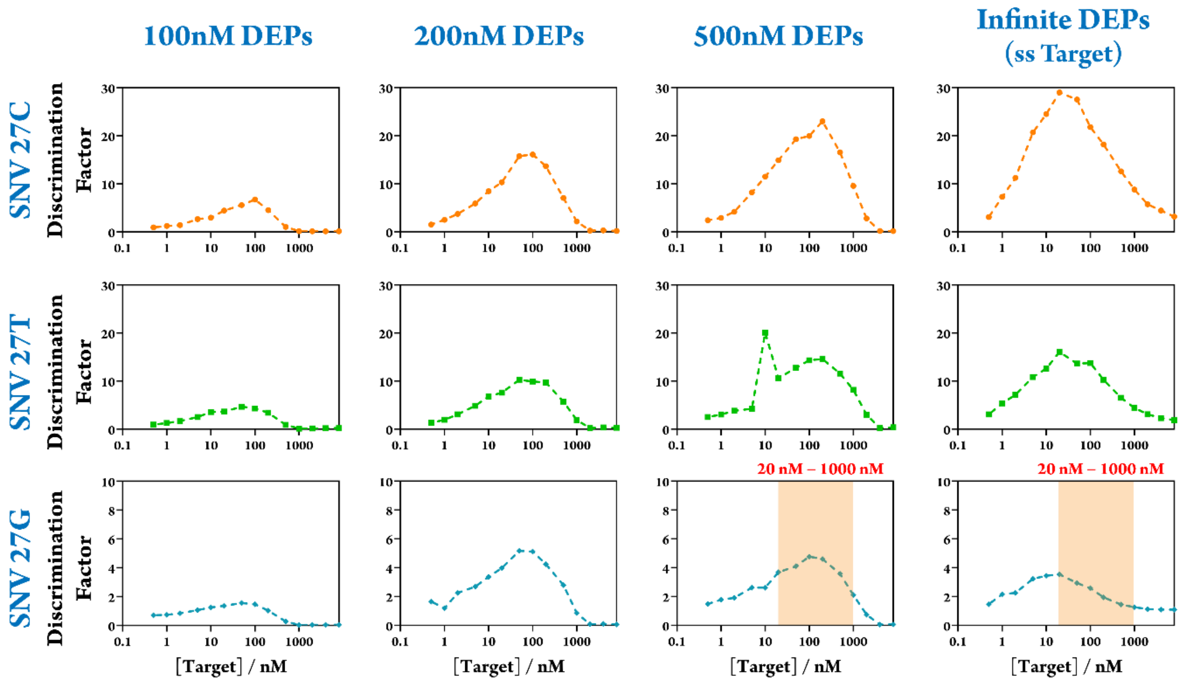

Supplementary Figure 29 | Experimentally measured DF values for three single nucleotide mutations (SNV27C, SNV27T, and SNV27G) in the 44 bp HBV target using DEG. Shaded area indicates the detection window where DEG outperforms toehold-exchange beacon in discriminating the most challenge SNV, 27G.

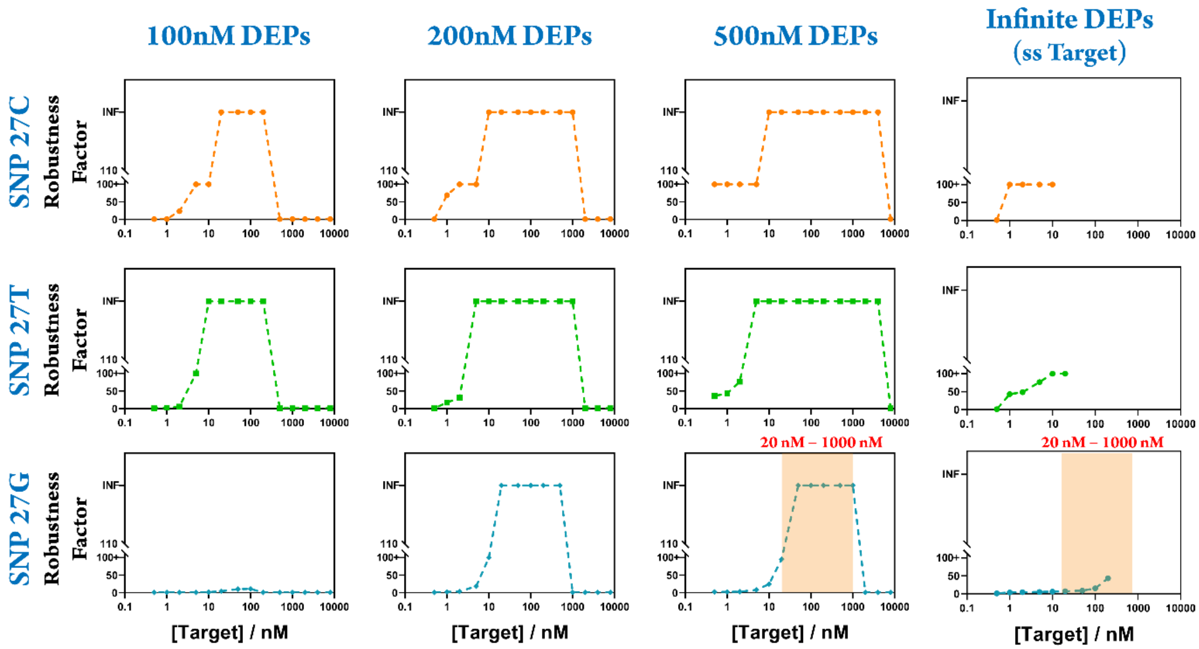

Supplementary Figure 30 | Experimentally measured RF values for single nucleotide mutations in the 44 bp HBV target using DEG.

5.3 Detection of clinically important single nucleotide variants in cancer

To further demonstrate the versatility and robustness of our DEG method, we designed 9 sets of DEG and toehold-exchange probes for clinically important single nucleotide variants frequently detected in cancer. The sequences and designs are shown in Supplementary Fig. 31 and the performance of DEG for analyzing the 9 sets of targets are shown in Supplementary Fig. 32 and 33.

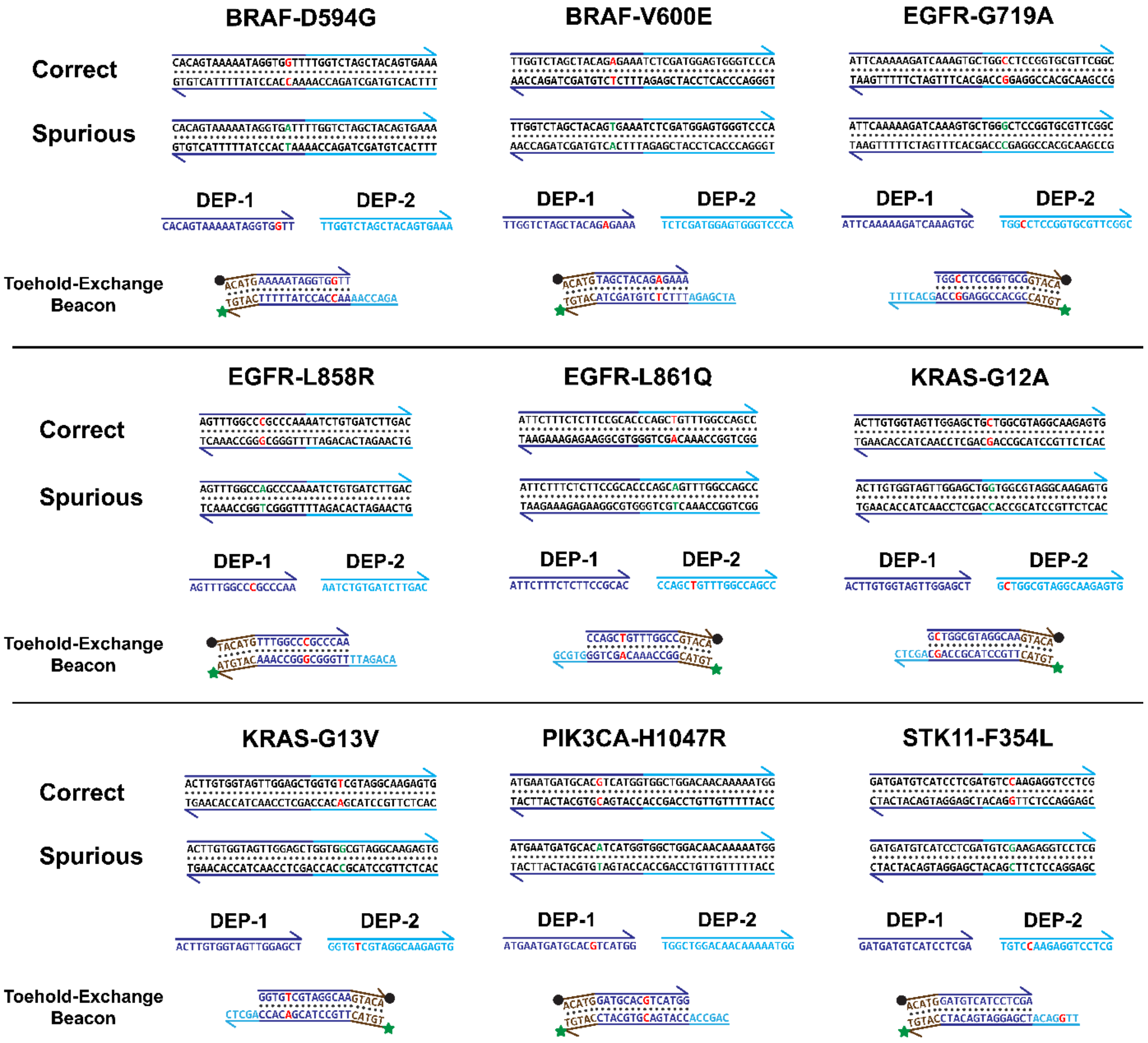

Supplementary Figure 31 | Designs and sequences for 9 sets of clinically important single nucleotide variants frequently encountered in cancer.

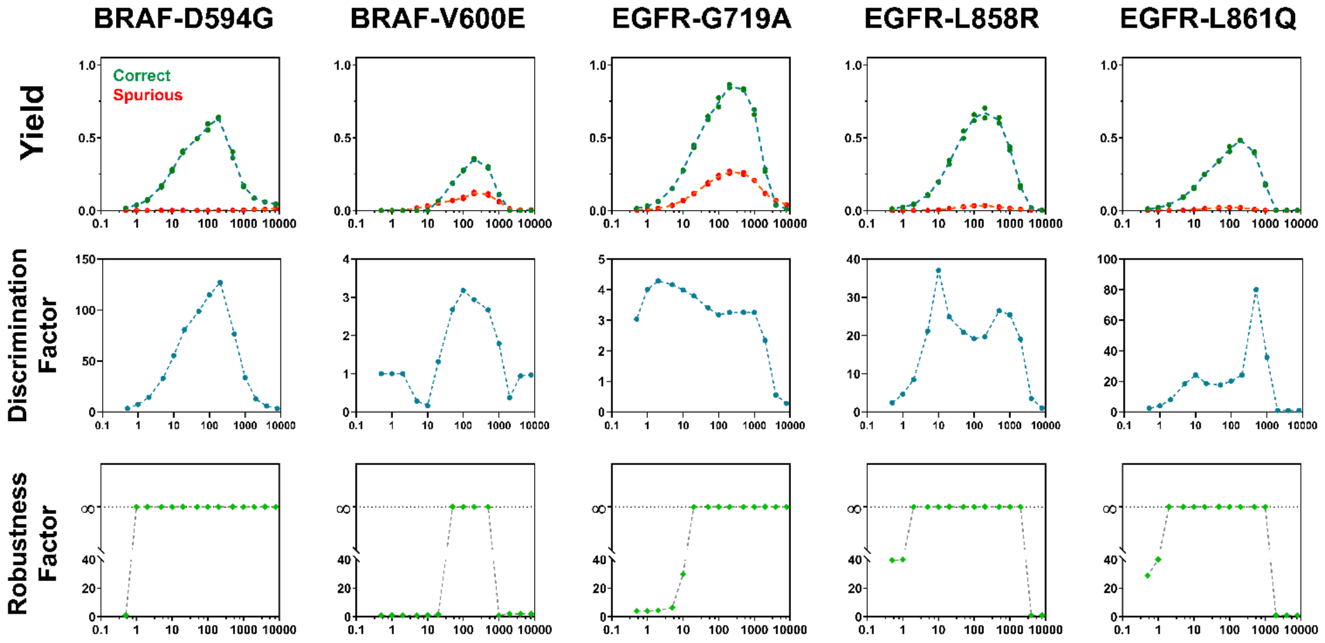

Supplementary Figure 32 | Experimentally measured yields, DFs, and RFs for analyzing BRAF-D594G, BRAF-V600E, EGFR-G719A, EGFR-L858R and EGFR-L861Q. The concentration of DEG is fixed at 200 nM. Values of individual replicates (n=2) of yield are shown as dots, and lines represent the mean values (correct and spurious curves).

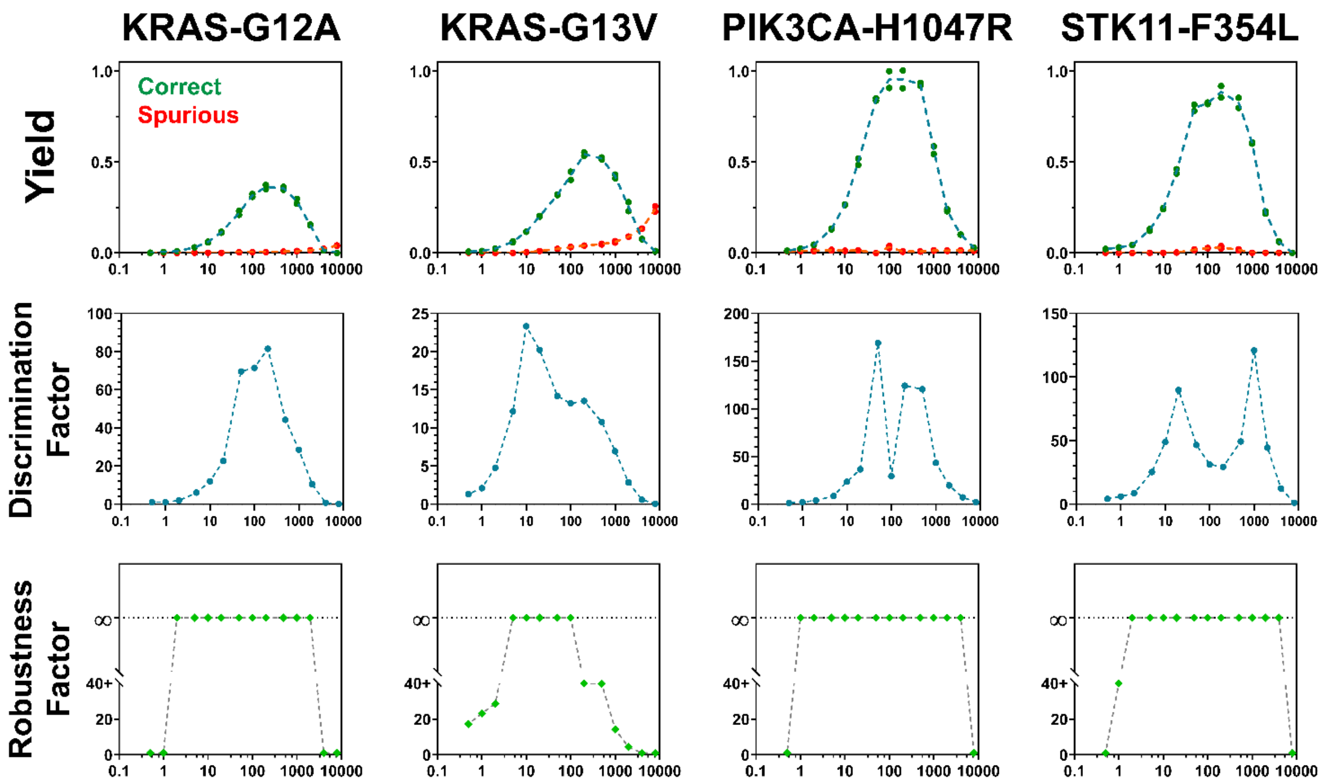

Supplementary Figure 33 | Experimentally measured yields, DFs, and RFs for analyzing KRAS-G12A, KRAS-G13V, PIK3CA-H1047R and STK11-F354L. The concentrations of DEPs were fixed at 200 nM. Values of individual replicates (n=2) of yield are shown as dots, and lines represent the mean values (correct and spurious curves).

6. Evaluation of DEG for detection of rare mutations

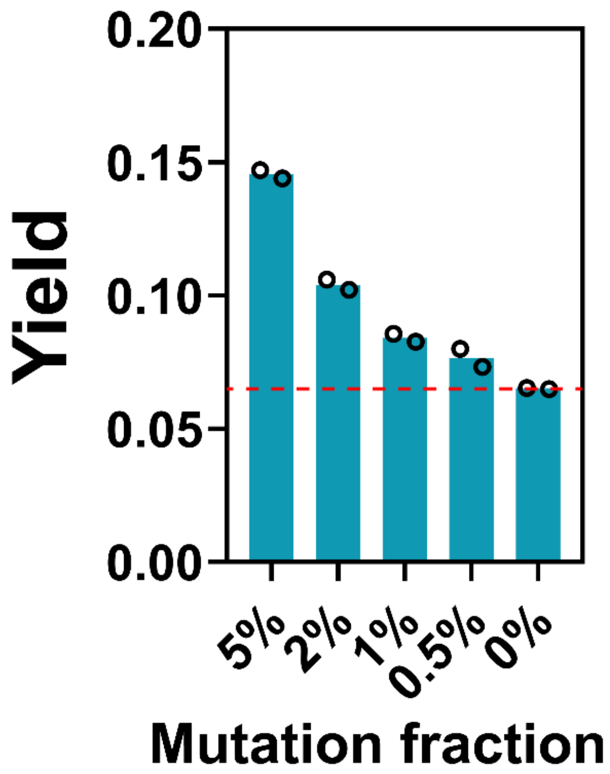

Supplementary Figure 34 | As low as 0.5% mutated targets in the background of high concentrations of unmutated sequences can be detected effectively using DEG. Bars represent the mean values of individual replicates (circles, n=2).

7. Multiplexity of DEG

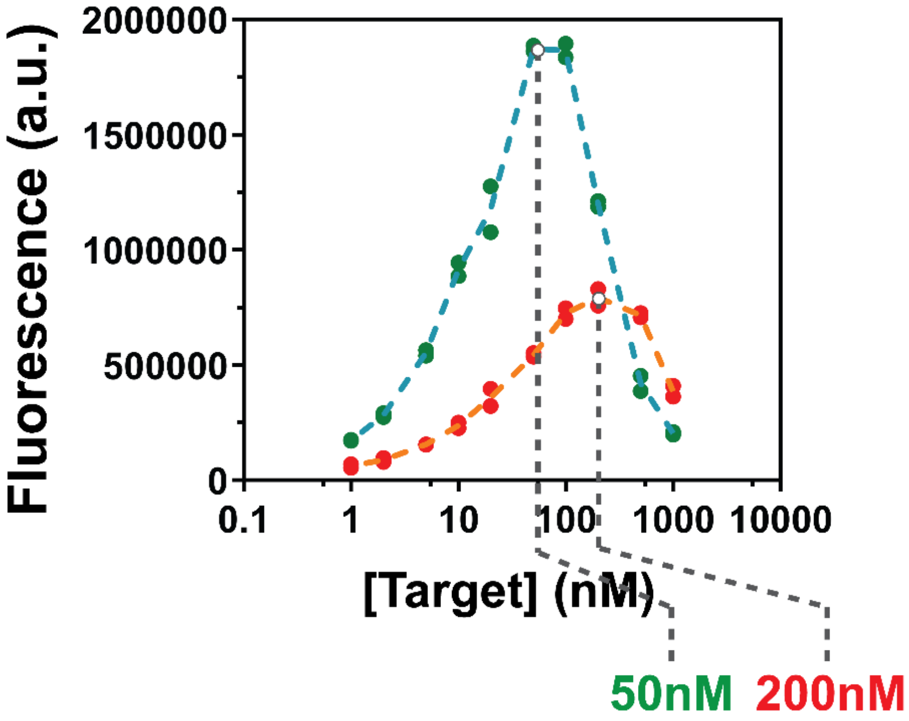

Supplementary Figure 35 | Simultaneous manipulation of TT and HBV targets using two sets of DEPs in the same test tube. The characteristic detection curves were observed for each target with detection window controlled by their corresponding DEPs ([DEP] = 50 nM for TT and 200 nM for HBV). This experiment demonstrates that multiplexed DEG can be performed for the independent control of multiple strand displacement reactions in the same test tube. Values of individual replicates (n=2) of yield are shown as dots, and lines represent the mean values.

8. DEG PCR

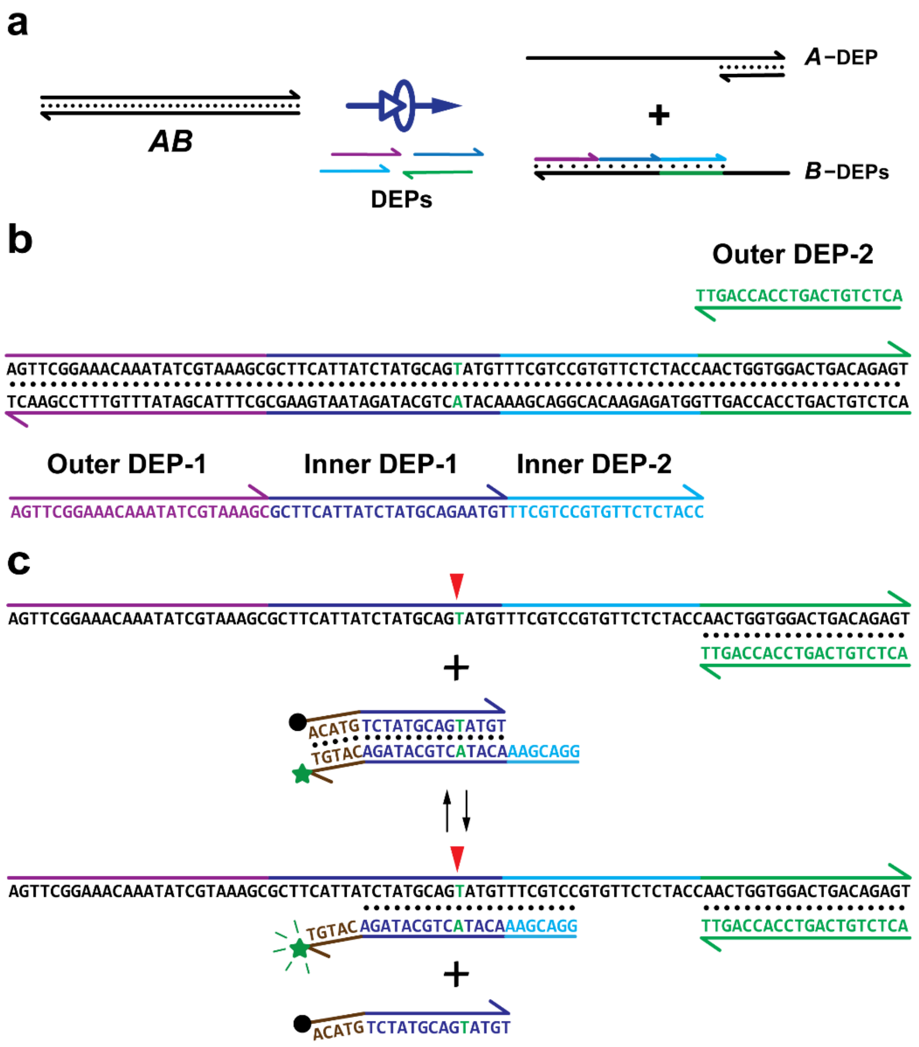

Supplementary Figure 36 | Schematic illustration of the sequence design for a set of four DEPs that target an 87 bp amplicon (AB) for the detection of the hotspot for drug resistance in TT worm. a. Scheme showing the 4-DEP design for the PCR amplicon. **b.** The two inner DEPs were designed to expose ssDNA domains that can be detected by the reporter probe. The two outer DEPs were designed to facilitate the generation of ssDNA output (A) the sequence of which were identical with a pair of forward and reverse PCR primers. **c.** The design of the reporter probe operated by the toehold-exchange.

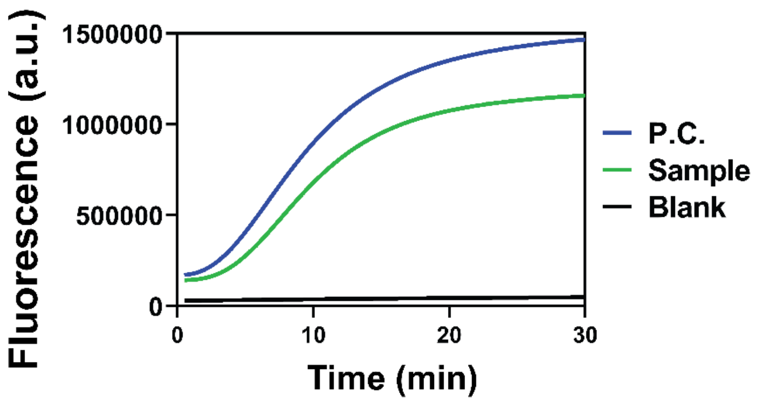

Supplementary Figure 37 | Validation of DEG for the detection of ssDNA output A *via* the 4-DEP design. Real-time fluorescence monitoring the kinetics of the reporter for measuring **A** produced by 4-DEPs equalizer gate. Concentrations of all DEPs were fixed at 200 nM for detection of 20 nM of target ***AB*** with drug resistance mutation (mutant), generating 80% of fluorescent yield. Our DEG approach allows the discrimination of single nucleotide mutation with high sequence specificity.

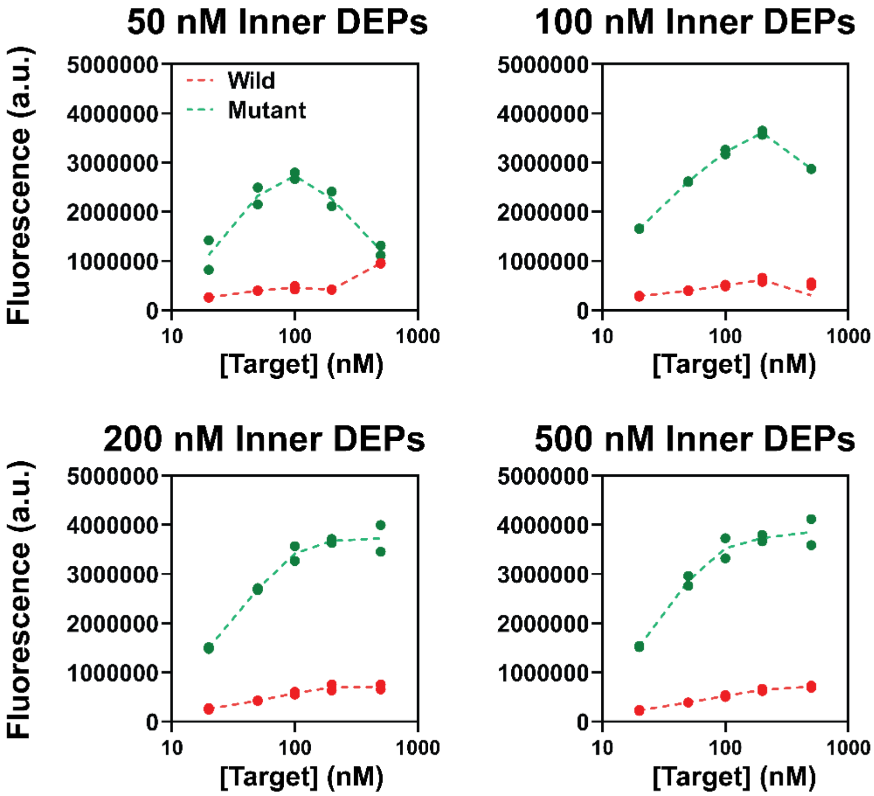

Supplementary Figure 38 | Analyzing PCR amplicons using DEG with varying concentrations of inner DEPs from 50 nM to 500 nM (500nM outer DEPs). DEG allows the detection of the double-stranded PCR amplicon meanwhile discrimination of single nucleotide mutation through wide concentration ranges. Lines represent the mean values of individual replicates (red and green dots, n=2).

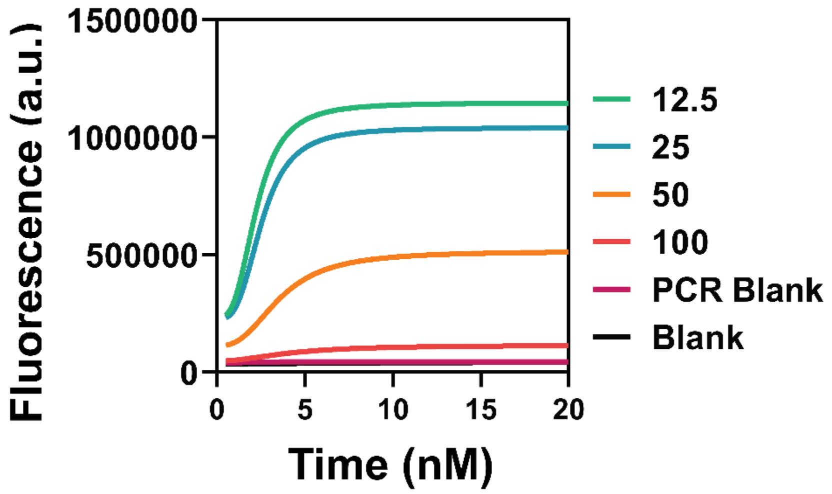

Supplementary Figure 39 | Optimization of the asymmetric PCR using varying ratios between the concentrations of forward and reverse primers. Kinetic curves showing the measurement of ssDNA output generated by asymmetric PCR using the reporter probe that is operated using toehold-exchange.

9. Analyzing clinical parasitic worm specimens using DEG-PCR

Infections caused by virus, bacteria, and parasitic worms are major threats to human worldwide. The extensive uses of antibiotics for treating various infectious diseases (often because of insufficient diagnosis) also leads to the issue of drug resistance. Therefore, an ideal test for diagnosing infectious diseases shall not only detect specific pathogens with high accuracy, it shall also screen or identify drug resistance and thus guide the treatment. Toward this goal, we engineered DEG-PCR by introducing a dual reporter system that allows the simultaneous detection of infections caused by *Trichuris trichiura* (TT) and screen for drug resistance. The first reporter (FAM-reporter) that is operated through the principle of toehold-exchange is designed to a specific A to T mutation at the 200th codon of β-tubulin, which is a well-established hotspot of TT for resistance to benzimidazole (BZ, drug). As such, the fluorescence of this reporter (FAM) is turned on only when drug resistance occurs in TT infection (D.R.+ plus TT infection). The second reporter (Cy5-reporter) that is operated by toehold-mediated strand displacement is designed to detect TT infection. The reporter has a reverse toehold of 0 and is thus not sensitive to single-nucleotide mutations, which ensures the detection of infections regardless of the existence of SNPs. Simultaneous detection of the two fluorescence channels (FAM and Cy5) allows the detection of infection and screen for drug resistance in a single test.

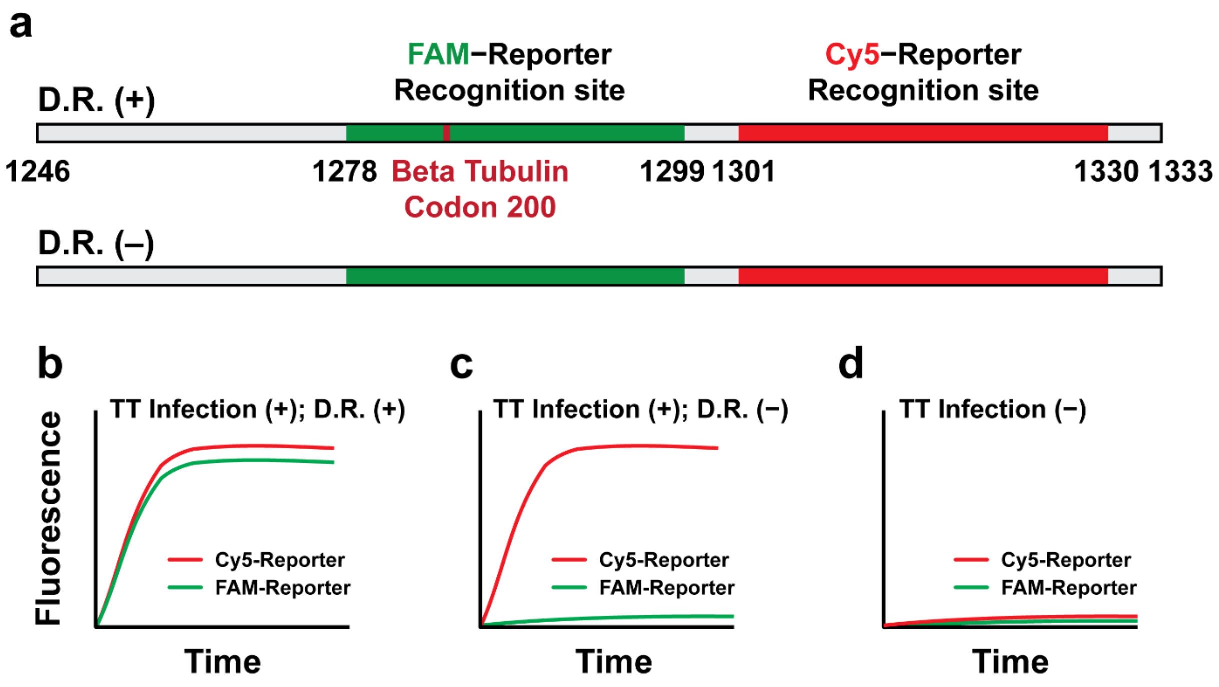

Supplementary Figure 40 | The sequence design of DEG-PCR for analyzing clinical parasitic worm samples. a. A pair of primers that amplify β-tubulin gene from 1246 to 1333 bp are designed through primer-design software BLAST. The FAM reporter (green) is designed to detect the specific A to T mutation at codon 200 at the 1278th to 1299th bp of the β-tubulin gene. The Cy5-reporter (red) is designed to analyze the 1301st to 1330th bp of the β-tubulin gene. **b.** The representative fluorescence kinetic curves that indicate positive infection (red) and positive drug resistance (green, D.R.+). **c**. The representative fluorescence kinetic curves that indicate positive infection (red) but negative drug resistance (green, D.R.–). **d**. The representative fluorescence kinetic curves that indicate no infection and no drug resistance.

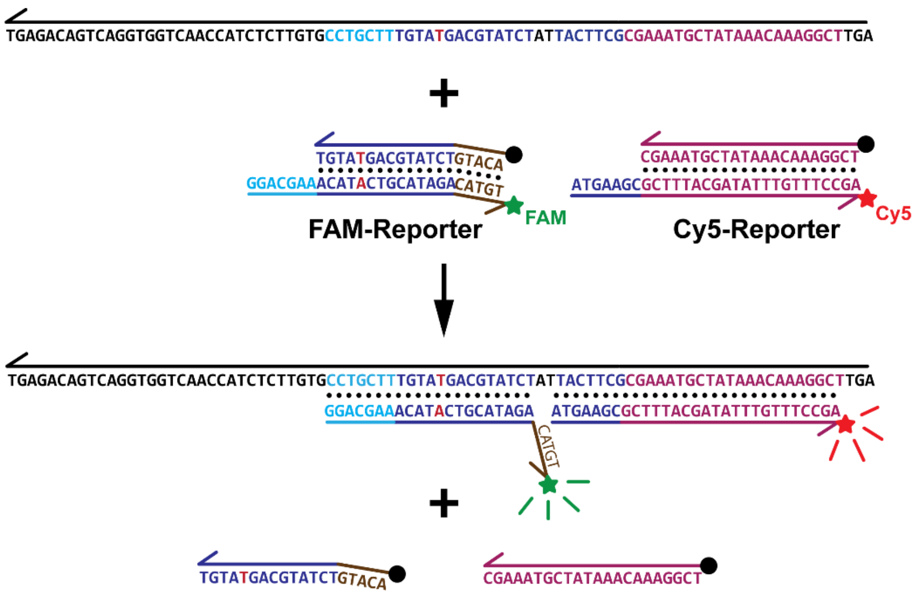

Supplementary Figure 41 | Schematic representation of the sequence designs for the DEPs and two reporters.

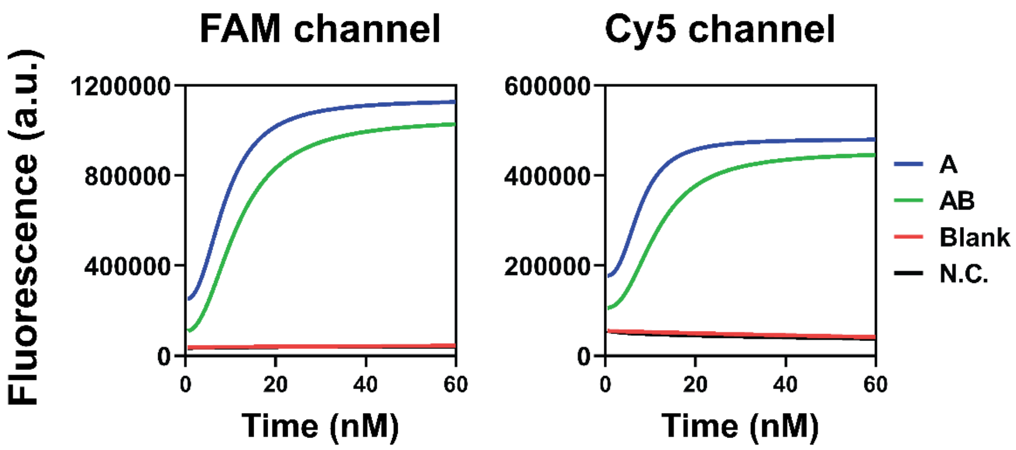

Supplementary Figure 42 | Validation of the 4-DEP, dual reporter DEG for the detection of the double-stranded TT target with the A to T mutation at β-tubulin codon 200. Real-time fluorescence monitoring the kinetics of both FAM- and Cy5-reporter for measuring the double-stranded TT target (AB) at two distinct sites as indicated in Supplementary Fig. 41. Rapid increases in fluorescence was observed in both channels when detecting 20 nM T using a set of 4 DEPs with 200 nM for each.

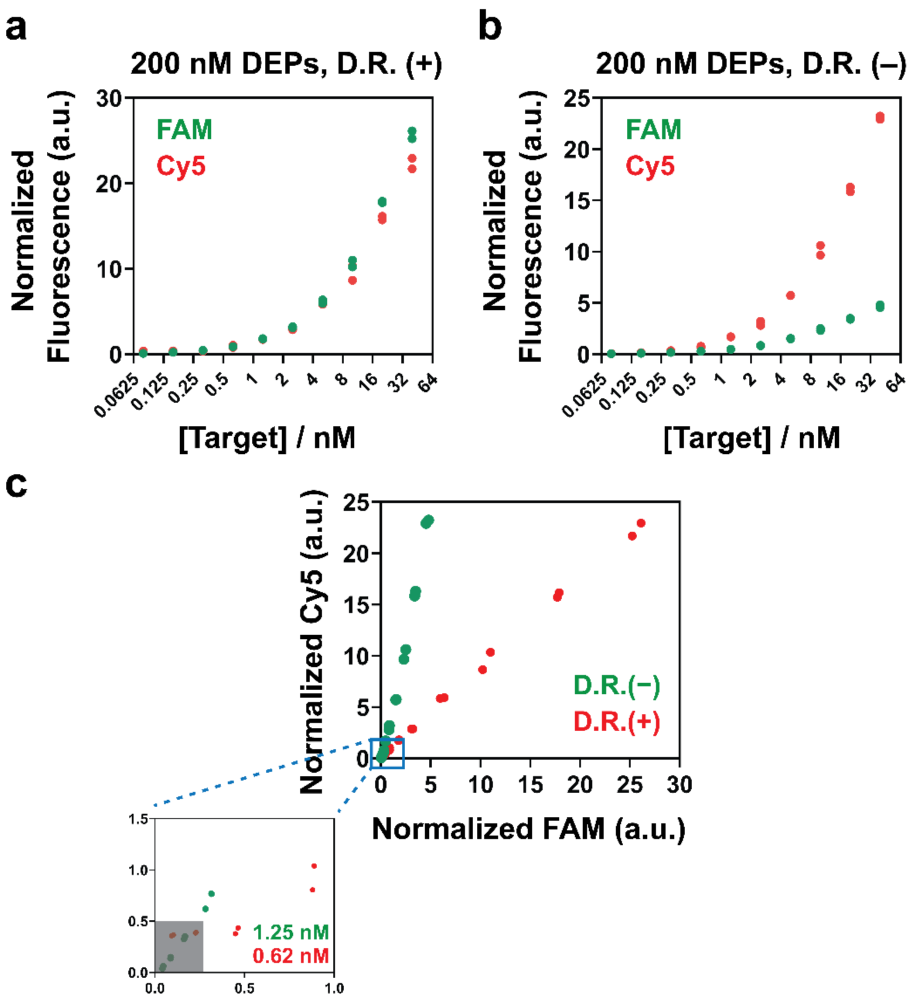

Supplementary Figure 43 | The detection limit of the 4-DEP, dual reporter DEG for analyzing synthetic DNA targets either drug resistant mutant that is drug resistant positive (D.R.+) or a wild-type that is drug resistant negative (D.R.–). a. Normalized fluorescence in both FAM and Cy5 channels as a function of target concentrations for the detection of drug resistant positive mutant. **b.** Normalized fluorescence in both FAM and Cy5 channels for the detection of a wild-type target. **c.** The dual-channel fluorescence distribution map for targets (either D.R.+ or D.R.–) spanning over 0.16, 0.31, 0.62, 1.25, 2.5, 5, 10, 20, 40, and 80 nM. The detection limit was found to be 0.62 nM for drug resistant positive target while 1.25 nM for drug resistant negative target. Gray shading area in the subplot indicating the

fluorescence distribution that cannot distinguish D.R+ and D.R.– targets. Values of individual replicates (n=2) are shown as dots.

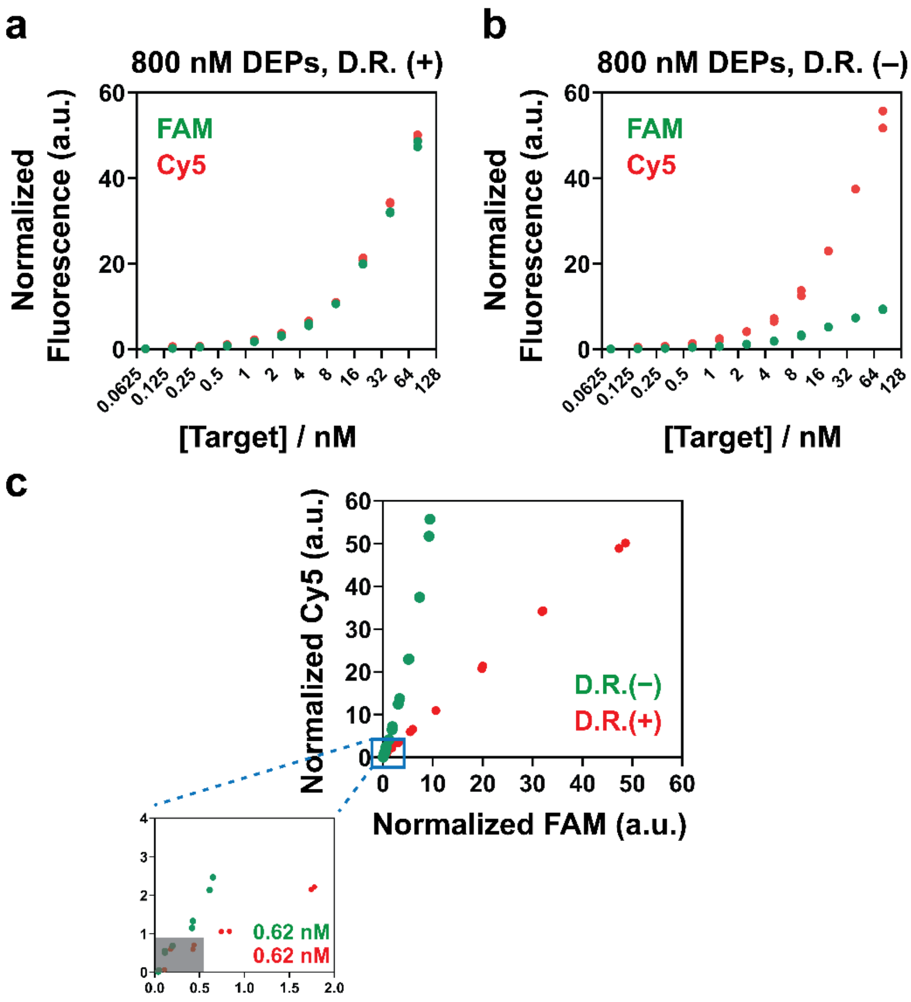

Supplementary Figure 44 | The detection limit of the 4-DEP, dual reporter DEG for analyzing synthetic DNA targets with 800 nM DEPs. a. Normalized fluorescence in both FAM and Cy5 channels as a function of target concentrations for the detection of drug resistant positive mutant. **b.** The fluorescence distribution of FAM and Cy5 channels as a function of mutant-type target concentration. **c.** The dual-channel fluorescence distribution map for targets (either D.R.+ or D.R.–) spanning over 0.16, 0.31, 0.62, 1.25, 2.5, 5, 10, 20, 40, and 80 nM. The detection limit was found to be 0.62 nM for both drug

resistant positive and drug resistant negative targets. Gray shading area in the subplot indicating the fluorescence distribution that cannot distinguish D.R+ and D.R.– targets. Values of individual replicates (n=2) are shown as dots.

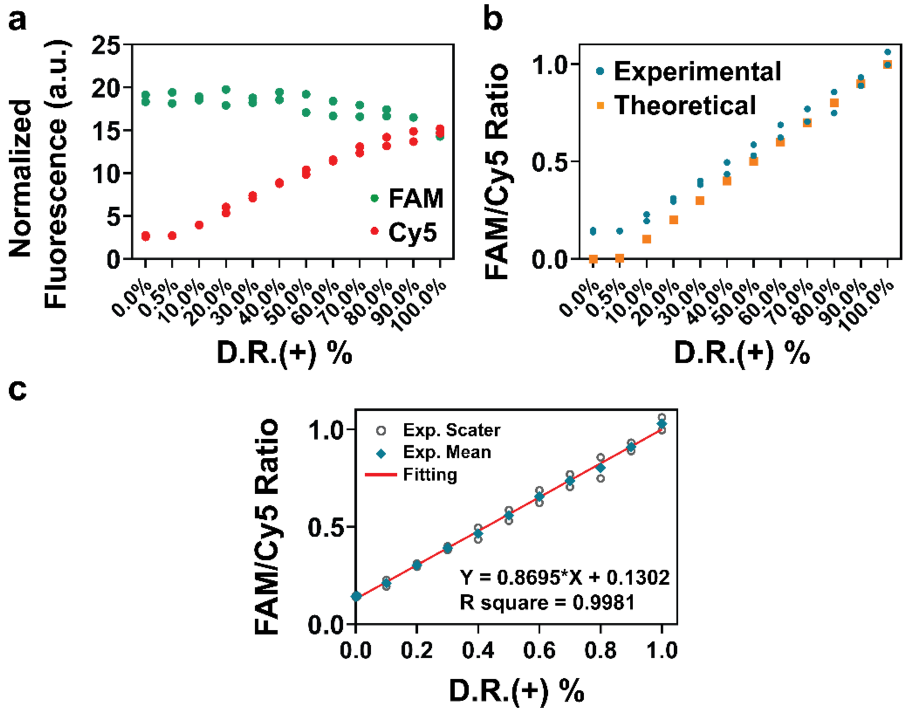

Supplementary Figure 45 | Detection of drug resistant mutant in the presence of varying concentrations of wild-types. a. Normalized fluorescent intensities of FAM and Cy5 channels as a function of the percentage of spiked mutant in the wild-type control. The total target concentration was fixed at 20 nM. Values of individual replicates (n=2) are shown as dots. **b.** Experimental (blue dots, n=2) and theoretical (yellow squares) calibration curves using FAM/Cy5 ratio as a readout. **c.** Linear regression of the experimental calibration mean values. This result suggests that our 4-DEP, dual reporter DEG approach was able to discriminate clinically important single nucleotide variants present at low abundance.

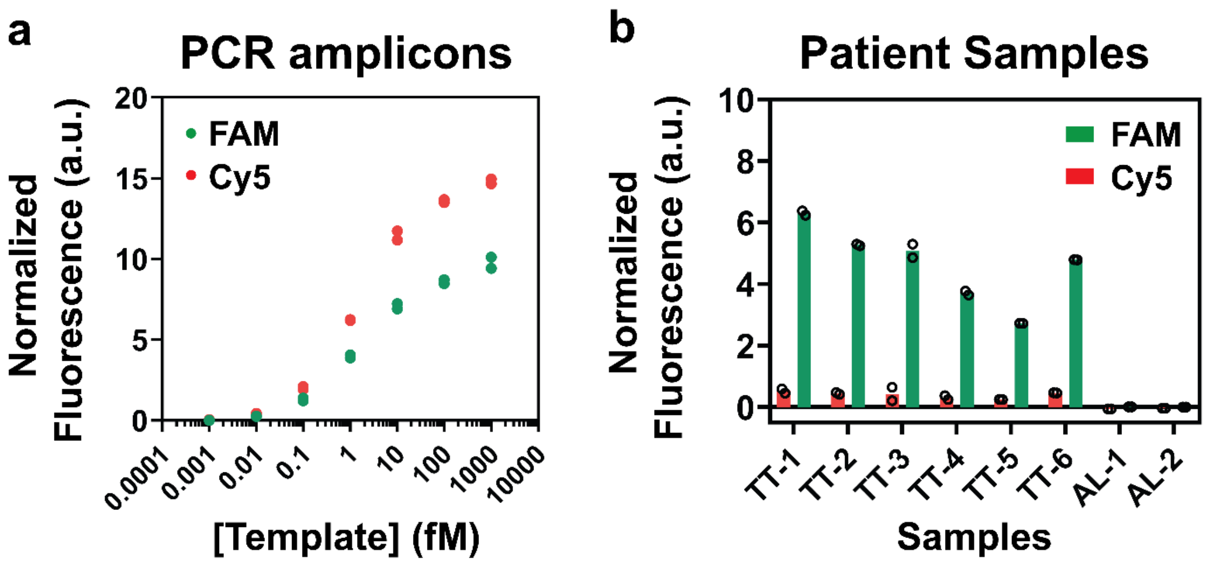

Supplementary Figure 46 | Deployment of the dual reporter DEG-PCR for analyzing clinical parasitic worm samples. a. Normalized fluorescent intensities of FAM and Cy5 channels as a function of the original concentrations of the synthetic DNA template prior to PCR amplification. This template is of the same subgenomic sequence as the drug-resistant mutate. Values of individual replicates (n=2) are shown as dots. **b.** Normalized fluorescence intensities of FAM and Cy5 channels for clinical parasitic worm samples, including 6 *Trichuris trichiura* (TT) and 2 *Ascaris Lumbricoides* (AL) worms. All the TT samples show positive infection but negative drug-resistance; while two AL samples are showing negative TT infection. The negative fluorescence intensity indicates that fluorescence signals for AL are lower than that for the blank. Bars represent the mean values of individual replicates (circles, n=2).

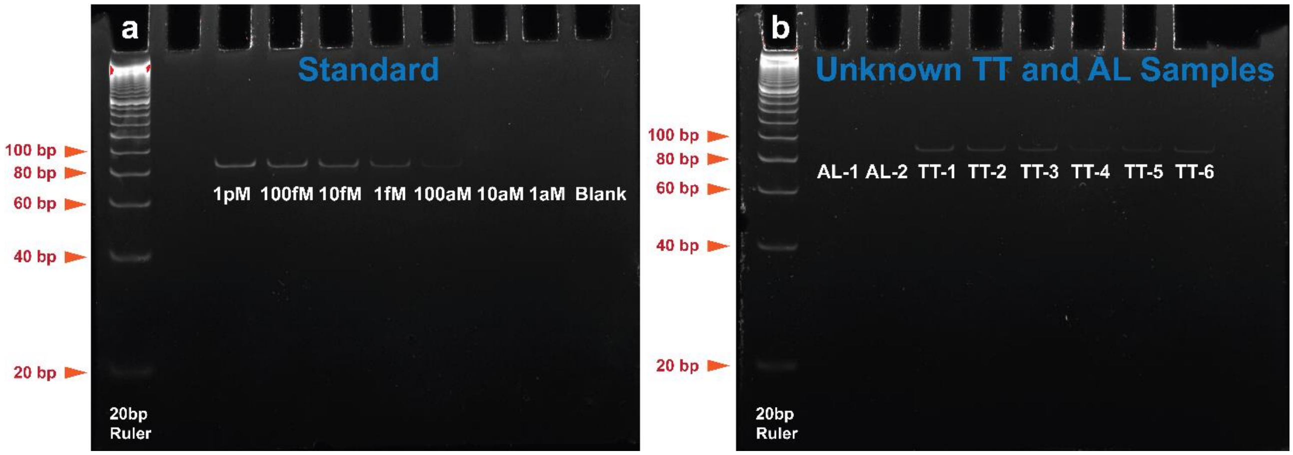

Supplementary Figure 47 | Detection of clinical parasitic worm samples using standard PCR followed by polyacrylamide gel electrophoresis (PAGE) analysis. a. Representative PAGE analysis of PCR amplicons of standard synthetic DNA templates ranging from 1 aM to 1 pM. **b**. Representative PAGE analysis of PCR amplicons of eight clinical parasitic worm samples. Each experiment was repeated twice to verify the reproducibility. A 5-µL solution containing PCR amplicons was mixed with loading buffer and then loaded onto 6% PAGE gel. A voltage of 110 V was applied for driving the electrophoresis. After electrophoresis, the gel was stained with Ethidium Bromide and imaged using Gel Doc XR+ Imager System (BioRad).

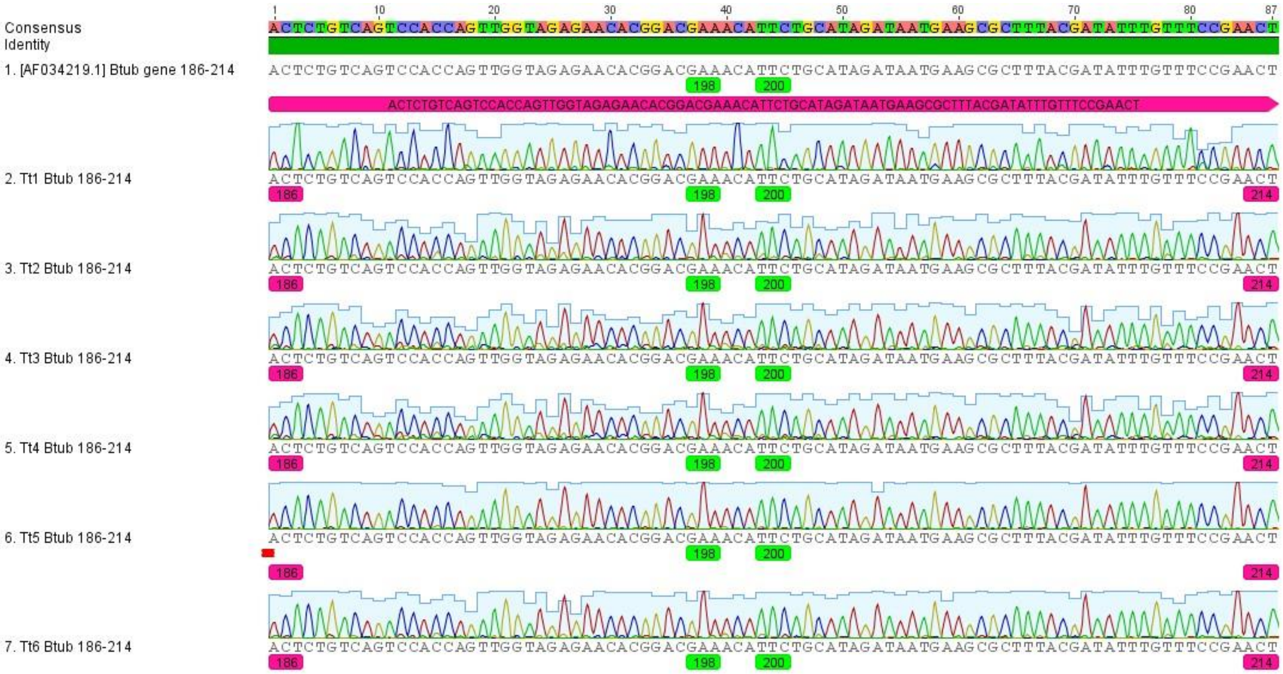

Supplementary Figure 48 | Genome sequencing data of clinical parasitic worms. The first row illustrates the DNA sequence of codon 186 to codon 214 for wild-type *Trichuris trichiura β*-tubulin gene. Codon 198 and 200 are highlighted as drug-resistance mutation hotspots. Six worm specimens extracted from patients were of the same sequences with the wild-type, which was well consistent with the diagnostic results measured using DEG-PCR.

10. DNA Sequences and Modifications

Supplementary Table 1. DNA sequences information.

DNA Names		Sequences
Synthetic target	Correct Target	5′– GC TTC AT TA TC TAT GCA GTA TGT TTC GTC C GT GTT CTC TAC C –3′
	Spurious Target	5′– GC TTC AT T A TC TAT GCA GAA TGT TTC GTC C GT GTT CTC TAC C –3′
	DEP-1	5′– GC TTC AT TA TC TAT GCA GTA TGT –3′
	DEP-2	5′– TTC GTC C_GT GTT CTC TAC C –3′
	Reporter-F	5′– G GAC GAA ACA TAC TGC ATA GA CATGT-FAM –3′
	Reporter-Q	5′– Iowa Blank FQ– ACATG TC TAT GCA GTA TGT –3′
HBV44	Correct Target	5′– AA ATT CGC AGT CCC CAA CCT CC AATC ACT CAC CAA CCT CCT GTC –3′
	HBV-DEP-1	5′– AA ATT CGC AGT CCC CAA CCT CC –3′
	HBV-DEP-2	5′– A ATC ACT CAC CAA CCT CCT GTC –3′
	SNV27T	5′– AA ATT CGC AGT CCC C AA CCT CC AATC TCT CAC CAA CCT CCT GTC –3′
	SNV27G	5′– AA ATT CGC AGT CCC C AA CCT CC AATC GCT CAC CAA CCT CCT GTC –3′
	SNV27C	5′– AA ATT CGC AGT CCC C AA CCT CC AATC CCT CAC CAA CCT CCT GTC –3′
	INS27A	5′– AA ATT CGC AGT CCC C AA CCT CC AATC AACT CAC CAA CCT CCT GTC –3′
	INS27C	5′– AA ATT CGC AGT CCC C AA CCT CC AATC CACT CAC CAA CCT CCT GTC –3′

	DEL27	5′- AA ATT CGC AGT CCC C AA CCT CC AATC CT CAC CAA CCT CCT GTC -3′
	Reporter-F	5′- FAM- CGCTT AGG TTG GTG AGT GATT GG AGG TT -3′
	Reporter-Q	5′- A ATC ACT CAC CAA CCT AAGCG -Iowa Black FQ -3′
TT42	Correct Target	5′- GC TTC AT TA TC TAT GCA GTA TGT TTC GTC C GT GTT CTC TAC C -3′
	SNV19A	5′- GC TTC AT T A TC TAT GCA GAA TGT TTC GTC C GT GTT CTC TAC C -3′
	SNV19G	5′- GC TTC AT T A TC TAT GCA GGA TGT TTC GTC C GT GTT CTC TAC C -3′
	SNV19C	5′- GC TTC AT T A TC TAT GCA GCA TGT * TTC GTC C GT GTT CTC TAC C -3′
TT42	TT-DEP-1	5′- GC TTC AT TA TC TAT GCA GTA TGT -3′
	TT-DEP-2	5′- TTC GTC CGT GTT CTC TAC C -3′
TT84	Correct Template (T^+)	5'- AGT TCG GAA ACA AAT ATC GTA AAG C GC TTC AT TA TC TAT GCA GAA TGT TTC GTC C GT GTT CTC TAC C AA CTG GTG GAC TGA CAG AGT -3'
	Spurious Template	5'- AGT TCG GAA ACA AAT ATC GTA AAG C GC TTC AT TA TC TAT GCA GTA TGT TTC GTC C GT GTT CTC TAC C AA CTG GTG GAC TGA CAG AGT -3'
	Forward Primer (TT-DEP-3)	5'- AGT TCG GAA ACA AAT ATC GTA AAG C -3'
	Reverse Primer (TT-DEP-4)	5'- ACT CTG TCA GTC CAC CAG TT -3'
	Cy5-reporter-F	5′- AT GAA GC G CTT TAC GAT ATT TGT TTC CGA -Cy5-3′

**Dual Channel TT Reporters**	**Cy5-reporter -Q**	5′- Iowa Blank RQ - TCG GAA ACA AAT ATC GTA AAG C -3′
	**FAM-Reporter-F**	5′- G GAC GAA ACA TAC TGC ATA GA CATGT-FAM -3′
	**FAM-Reporter-Q**	5′- Iowa Blank FQ- ACATG TC TAT GCA GTA TGT -3′

**TT87**	**Long-DEP-1**	5'-AGT TCG GAA ACA AAT ATC GTA AAG C GC TTC AT TA TC TAT GCA GTA TGT-3'
	**Long-DEP-2**	5′-TTC GTC CGT GTT CTC TAC C AAC TGG TGG ACT GAC AGA GT-3′
**TT62**	**Correct Target**	5'-ATC GTA AAG C GC TTC AT TA TC TAT GCA GTA TGT TTC GTC C_GT GTT CTC TAC C AAC TGG TGG A-3'
	**Spurious Target**	5'-ATC GTA AAG C GC TTC AT TA TC TAT GCA GAA TGT TTC GTC C_GT GTT CTC TAC C AAC TGG TGG A-3'
	**Long-DEP-1**	5'-ATC GTA AAG C GC TTC AT TA TC TAT GCA GTA TGT-3'
	**Long-DEP-2**	5′-TTC GTC CGT GTT CTC TAC C AAC TGG TGG A-3′
**TT32**	**Correct Target**	5'-TA TC TAT GCA GTA TGT TTC GTC C_GT GTT CTC T-3'
	**Spurious Target**	5'-TA TC TAT GCA GAA TGT TTC GTC C_GT GTT CTC T-3'
	**DEP-1**	5'-TA TC TAT GCA GTA TGT-3'
	**DEP-2**	5′-TTC GTC CGT GTT CTC T-3′
**TT28**	**SNV 1A**	5'-AC TAT GCA GTA TGT TTC GTC C_GT GTT CT-3'

TT28	SNV 1C	5'-CC TAT GCA GTA TGT TTC GTC C_GT GTT CT-3'
	SNV1G	5'-GC TAT GCA GTA TGT TTC GTC C_GT GTT CT-3'
	SNV 6T	5'-TC TAT TCA GTA TGT TTC GTC C_GT GTT CT-3'
	SNV 6C	5'-TC TAT CCA GTA TGT TTC GTC C_GT GTT CT-3'
	SNV 6A	5'-TC TAT ACA GTA TGT TTC GTC C_GT GTT CT-3'
	SNV 14A	5'-TC TAT GCA GTA TGA TTC GTC C_GT GTT CT-3'
	SNV 14C	5'-TC TAT GCA GTA TGC TTC GTC C_GT GTT CT-3'
	SNV 14G	5'-TC TAT GCA GTA TGG TTC GTC C_GT GTT CT-3'
	SNV 17T	5'-TC TAT GCA GTA TGT TTT GTC C_GT GTT CT-3'
	SNV 17A	5'-TC TAT GCA GTA TGT TTA GTC C_GT GTT CT-3'
	SNV 17G	5'-TC TAT GCA GTA TGT TTG GTC C_GT GTT CT-3'
BRAF-D594G	Correct Target	5'- CAC AGT AAA AAT AGG TGG TT | TTG GTC TAG CTA CAG TGA AA -3'
	Spurious Target	5'- CAC AGT AAA AAT AGG TGA TT | TTG GTC TAG CTA CAG TGA AA -3'
	DEP-1	5'- CAC AGT AAA AAT AGG TGG TT| -3'
	DEP-2	5'- TTG GTC TAG CTA CAG TGA AA| -3'

BRAF-D594G	TEB-F	5'- AGA CCA A | AA CCA CCT ATT TTT * CATGT -3'-36-FAM
	TEB-Q	5IABkFQ-5'- ACATG * AAA AAT AGG TGG TT| -3'
BRAF-V600E	Correct Target	5'- TTG GTC TAG CTA CAG AGA AA | T CTC GAT GGA GTG GGT CCC A -3'
	Spurious Target	5'- TTG GTC TAG CTA CAG TGA AA | T CTC GAT GGA GTG GGT CCC A -3'
	DEP-1	5'- TTG GTC TAG CTA CAG AGA AA| -3'
	DEP-2	5'- |T CTC GAT GGA GTG GGT CCC A -3'
	TEB-F	5'- ATC GAG A | TT TCT CTG TAG CTA * CATGT -3'-36-FAM
	TEB-Q	5IABkFQ -5'- ACATG * TAG CTA CAG AGA AA| -3'
EGFR-G719A	Correct Target	5'- ATT CAA AAA GAT CAA AGT GC | T GGC CTC CGG TGC GTT CGG C -3'
	Spurious Target	5'- ATT CAA AAA GAT CAA AGT GC | T GGG CTC CGG TGC GTT CGG C -3'
	DEP-1	5'- ATT CAA AAA GAT CAA AGT GC| -3'
	DEP-2	5'- |T GGC CTC CGG TGC GTT CGG C -3'
	TEB-F	56-FAM -5'- TGTAC * CGC ACC GGA GGC CA | G CAC TTT -3'
	TEB-Q	5'- |T GGC CTC CGG TGC G * GTACA -3'-3IABkFQ
EGFR-L858R	Correct Target	5'- AGT TTG GCC CGC CCA A | AA TCT GTG ATC TTG AC -3'

EGFR-L858R	Spurious Target	5'- AGT TTG GCC AGC CCA A | AA TCT GTG ATC TTG AC -3'
	DEP-1	5'- AGT TTG GCC CGC CCA A| -3'
	DEP-2	5'- |AA TCT GTG ATC TTG AC -3'
	TEB-F	5'- *ACA GAT T* | TT GGG CGG GCC AAA * *CATGT A* -3'-36-FAM
	TEB-Q	5IABkFQ -5'- *T ACATG* * T TTG GCC CGC CCA A| -3'
EGFR-L861Q	Correct Target	5'- ATT CTT TCT CTT CCG CAC | CCA GCT GTT TGG CCA GCC -3'
	Spurious Target	5'- ATT CTT TCT CTT CCG CAC | CCA GCA GTT TGG CCA GCC -3'
	DEP-1	5'- ATT CTT TCT CTT CCG CAC | -3'
	DEP-2	5'- |CCA GCT GTT TGG CCA GCC -3'
	TEB-F	56-FAM -5'- *TGTAC* * GGC CAA ACA GCT GG | *G TGC G* -3'
	TEB-Q	5'- CCA GCT GTT TGG CC * *GTACA* -3'-3IABkFQ
KRAS-G12A	Correct Target	5'- ACT TGT GGT AGT TGG AGC TGC TGG CGT AGG CAA GAG TG -3'
	Spurious Target	5'- ACT TGT GGT AGT TGG AGC TGG TGG CGT AGG CAA GAG TG -3'
	DEP-1	5'- ACT TGT GGT AGT TGG AGC T| -3'
	DEP-2	5'- |GCT GGC GTA GGC AAG AGT G -3'
	TEB-F	56-FAM -5'- *TGTAC* * TTG CCT ACG CCA GC | *A GCT C* -3'

KRAS-G12A	TEB-Q	5'- GCT GGC GTA GGC AA * *GTACA* -3'-3IABkFQ
KRAS-G13V	Correct Target	5'- ACT TGT GGT AGT TGG AGC TGG TGT CGT AGG CAA GAG TG -3'
	Spurious Target	5'- ACT TGT GGT AGT TGG AGC TGG TGG CGT AGG CAA GAG TG -3'
	DEP-1	5'- ACT TGT GGT AGT TGG AGC T| -3'
	DEP-2	5'- |GGT GTC GTA GGC AAG AGT G -3'
	TEB-F	56-FAM -5'- *TGTAC* * TTG CCT ACG ACA CC | *A GCT C* -3'
	TEB-Q	5'- GGT GTC GTA GGC AA * *GTACA* -3'-3IABkFQ
PIK3CA-H1047R	Correct Target	5'-ATG AAT GAT GCA CGT CAT GGT GGC TGG ACA ACA AAA ATG G -3'
	Spurious Target	5'-ATG AAT GAT GCA CAT CAT GGT GGC TGG ACA ACA AAA ATG G -3'
	DEP-1	5'- ATG AAT GAT GCA CGT CAT GG -3'
	DEP-2	5'- TGG CTG GAC AAC AAA AAT GG -3'
	TEB-F	5'- *CA GCC A* * CC ATG ACG TGC ATC * *CATGT* -3'-36-FAM
	TEB-Q	5IABkFQ -5'- *ACATG* * GAT GCA CGT CAT GG -3'
STK11-F354L	Correct Target	5'- GAT GAT GTC ATC CTC GAT GTC CAA GAG GTC CTC G -3'
	Spurious Target	5'- GAT GAT GTC ATC CTC GAT GTC GAA GAG GTC CTC G -3'
	DEP-1	5'- GAT GAT GTC ATC CTC GA -3'

STK11-F354L	DEP-2	5'- TGT CCA AGA GGT CCT CG -3'
	TEB-F	5'- *TTG GAC A* * TC GAG GAT GAC ATC * *CATGT* -3'-36-FAM
	TEB-Q	5IABkFQ -5'- *GTACA* * GAT GTC ATC CTC GA -3'

References

1. D. Y. Zhang, E. Winfree, Control of DNA Strand Displacement Kinetics Using Toehold Exchange. *J. Am. Chem. Soc.* **131**, 17303–17314 (2009).
2. S. X. Chen, D. Y. Zhang, G. Seelig, Conditionally fluorescent molecular probes for detecting single base changes in double-stranded DNA. *Nat. Chem*. **5**, 782–789 (2013).
3. J. Das, I. Ivanov, E. H. Sargent, S. O. Kelley, DNA Clutch Probes for Circulating Tumor DNA Analysis. *J. Am. Chem. Soc.* **138**, 11009–11016 (2016).
4. R. Lopez, R. Wang, G. Seelig, A molecular multi-gene classifier for disease diagnostics. *Nat. Chem*. **10**, 746–754 (2018).
