# Supplementary material for: Expanding detection windows for discriminating single nucleotide variants using rationally designed DNA equalizer probes
Source: Nat Commun. 2020 Oct 29;11:5473. doi: 10.1038/s41467-020-19269-9 (PMC7596233; doi:10.1038/s41467-020-19269-9)
Supplement: Supplementary file 2 — Reporting Summary [file 41467_2020_19269_MOESM2_ESM.pdf]

nature research

Corresponding author(s): Feng Li

Last updated by author(s): Sep 24, 2020

Reporting Summary

Nature Research wishes to improve the reproducibility of the work that we publish. This form provides structure for consistency and transparency in reporting. For further information on Nature Research policies, see our Editorial Policies and the Editorial Policy Checklist.

Statistics

For all statistical analyses, confirm that the following items are present in the figure legend, table legend, main text, or Methods section.

n/a	Confirmed	
☐	☒	The exact sample size (n) for each experimental group/condition, given as a discrete number and unit of measurement
☐	☒	A statement on whether measurements were taken from distinct samples or whether the same sample was measured repeatedly
☒	☐	The statistical test(s) used AND whether they are one- or two-sided<br>*Only common tests should be described solely by name; describe more complex techniques in the Methods section.*
☒	☐	A description of all covariates tested
☒	☐	A description of any assumptions or corrections, such as tests of normality and adjustment for multiple comparisons
☐	☒	A full description of the statistical parameters including central tendency (e.g. means) or other basic estimates (e.g. regression coefficient) AND variation (e.g. standard deviation) or associated estimates of uncertainty (e.g. confidence intervals)
☒	☐	For null hypothesis testing, the test statistic (e.g. F, t, r) with confidence intervals, effect sizes, degrees of freedom and P value noted<br>*Give P values as exact values whenever suitable.*
☒	☐	For Bayesian analysis, information on the choice of priors and Markov chain Monte Carlo settings
☒	☐	For hierarchical and complex designs, identification of the appropriate level for tests and full reporting of outcomes
☒	☐	Estimates of effect sizes (e.g. Cohen's d, Pearson's r), indicating how they were calculated

Our web collection on statistics for biologists contains articles on many of the points above.

Software and code

Policy information about availability of computer code

Data collection	The raw fluorescence data was collected by SpectraMax i3, along with SoftMax Pro 6.3 software.
Data analysis	The raw fluorescence data was analyzed by software Excel 2016, GraphPad Prism 8.0.1, and Matlab 2019a; thermodynamic data was estimated by free software NUPACK, http://www.nupack.org/partition/new. Source code for Matlab is available on Github, https://github.com/Crown1983/DNA-Equilizer-Gate.

For manuscripts utilizing custom algorithms or software that are central to the research but not yet described in published literature, software must be made available to editors and reviewers. We strongly encourage code deposition in a community repository (e.g. GitHub). See the Nature Research guidelines for submitting code & software for further information.

Data

Policy information about availability of data

All manuscripts must include a data availability statement. This statement should provide the following information, where applicable:

- Accession codes, unique identifiers, or web links for publicly available datasets
- A list of figures that have associated raw data
- A description of any restrictions on data availability

The raw fluorescence data of Figures 5, 6, 7, 8 in article and S18, S19, S21, S28, S32-S35, and S46 is available in Source Data package.

Field-specific reporting

Please select the one below that is the best fit for your research. If you are not sure, read the appropriate sections before making your selection.

☒ Life sciences ☐ Behavioural & social sciences ☐ Ecological, evolutionary & environmental sciences

For a reference copy of the document with all sections, see nature.com/documents/nr-reporting-summary-flat.pdf

Life sciences study design

All studies must disclose on these points even when the disclosure is negative.

Sample size	No sample size calculation was performed. Particularly, the patient sample size (8) used in this study is consistent with previous work (Ref. 42)
Data exclusions	no data exclusions
Replication	all technical replications (n=2) were successfully performed for all samples, as shown in Source Data file.
Randomization	Randomization was not relevant to our study. Toehold-exchange reaction had been well studied and deterministic mathematical models were build. In particular, simulation in this study was also based on deterministic equilibrium equations. To enhance the reproducibility and versatility of our DEG method, 9 clinically relevant targets (Fig. S31-S33) were arbitrarily chosen without previous study.
Blinding	Blinding was not applied in this study. The aim of this work is to develop a class of hybridization probes to expand detection window of SNVs, which is based on well studied toehold-exchange reaction and deterministic simulations.

Reporting for specific materials, systems and methods

We require information from authors about some types of materials, experimental systems and methods used in many studies. Here, indicate whether each material, system or method listed is relevant to your study. If you are not sure if a list item applies to your research, read the appropriate section before selecting a response.

Materials & experimental systems

n/a	Involved in the study	
☒	☐	Antibodies
☒	☐	Eukaryotic cell lines
☒	☐	Palaeontology and archaeology
☒	☐	Animals and other organisms
☐	☒	Human research participants
☒	☐	Clinical data
☒	☐	Dual use research of concern

Methods

n/a	Involved in the study	
☒	☐	ChIP-seq
☒	☐	Flow cytometry
☒	☐	MRI-based neuroimaging

Human research participants

Policy information about studies involving human research participants

Population characteristics	School-age children in the rural region of La Hicaca located in the northwestern area of Honduras were invited to enroll in the study.
Recruitment	Upon parents or guardians' consent, children were invited to enroll in the study and those willing to participate provided verbal assents that were documented on a child assent form with the signature of a third-party witness. A subgroup of eight children harboring infections of heavy and moderate intensity were invited to receive a special deworming treatment in order to recover adult parasite specimens.
Ethics oversight	This study received clearance from the Bioscience Research Ethics Board of the Brock University (file number 13-195), as well as from the Research Ethics Board of the master's Program in Infectious and Zoonotic Diseases (MEIZ), School of Microbiology, National Autonomous University of Honduras (UNAH).

Note that full information on the approval of the study protocol must also be provided in the manuscript.
